# Supplementary material for: Phase information is conserved in sparse, synchronous population-rate-codes via phase-to-rate recoding
Source: Nat Commun. 2023 Sep 30;14:6106. doi: 10.1038/s41467-023-41803-8 (PMC10543394; doi:10.1038/s41467-023-41803-8)
Supplement: Supplementary file 1 — Supplementary Information [file 41467_2023_41803_MOESM1_ESM.pdf]

Supplementary Information for

Phase information is conserved in sparse, synchronous population-rate-codes via phase-to-rate recoding by Müller-Komorowska et al., 2023.

Supplementary Figures

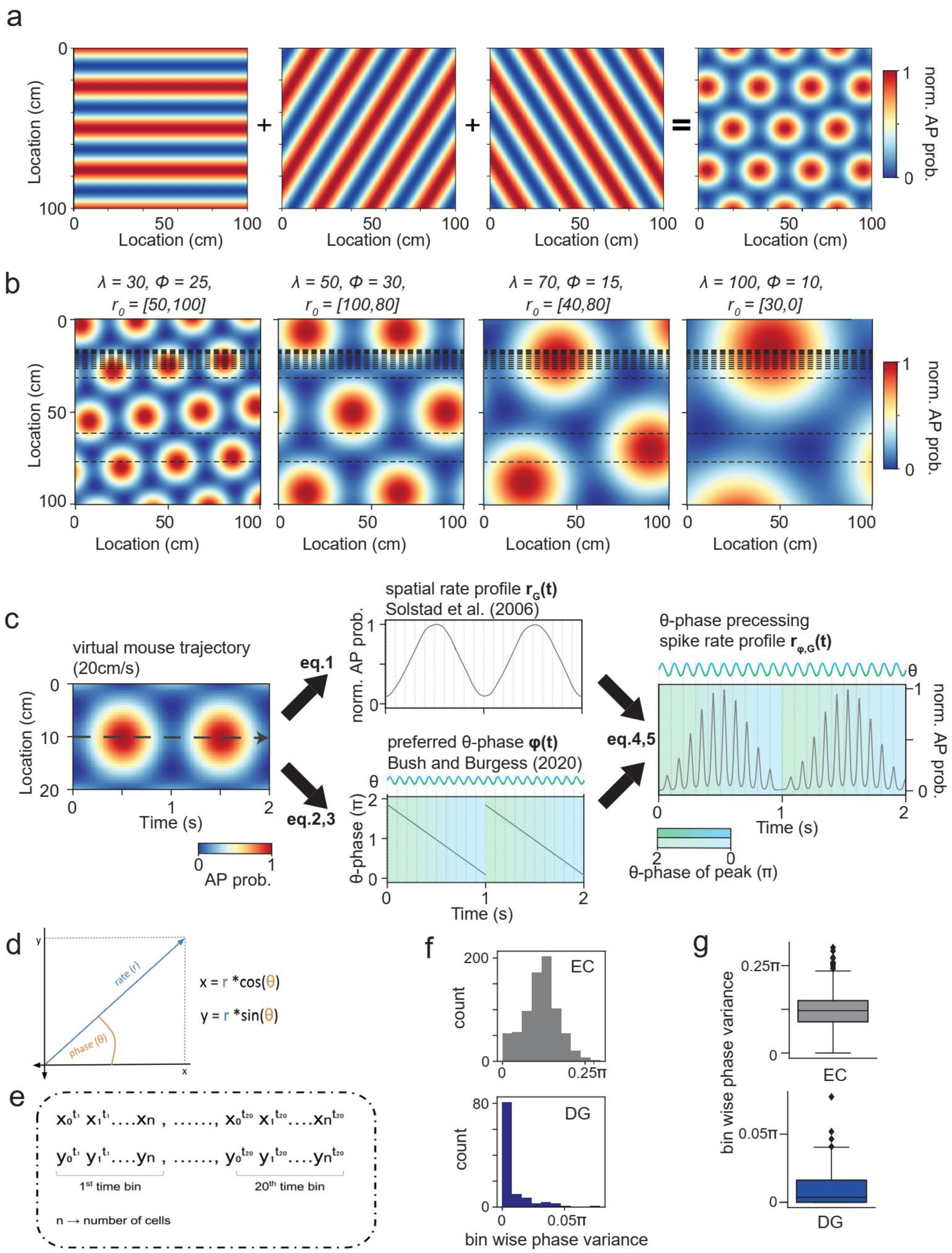

Supplementary Figure 1. Phenomenological grid cell phase precession model. Phase precessing grid cell inputs were created by combining a grid cell model[1] and a phase precession[2] model. **a** Grid field firing patterns were created as in [1] by overlaying three sinusoidal gratings. **b** Four example grid cells and the simulated linear trajectories (black dashed lines). To generate a population of grid cells, grid cell parameters were matched to empirical observations[3]. **c** Illustration of how grid model and phase precession model were combined. We assumed linear trajectory through virtual space and a constant theta oscillation yielding i) a spatial rate profile (eq.1) and ii) a preferred firing phase for each location (eq.2). The latter was then converted into a sequence of Gaussian-like probability distribution with their peaks at the respective preferred phases (eq. 4), and this probability distribution was multiplied with the spatial rate profile (eq.5). The combined probability distribution now defines a phase-precessing grid-cell code, that could be calculated for each respective grid cell. It was used as the basis of an inhomogeneous Poisson process to generate spiking patterns. **d** Translation of polar to Cartesian coordinates was performed for each cell and theta-cycle based on the respective mean values (this avoids the issue of undefined phases when there are no spikes). **e** This yields 2 values (*x,y*) per cell and theta-cycle. **f** Distribution of phase variance of spikes within individual theta bins (for the full network, 40 randomly chosen cells, and 20 Poisson seeds). **G** Same data as f. Mean variances within a theta-cycle were 21±10° and 2±3° for EC-grid and DG-granule cells, respectively.

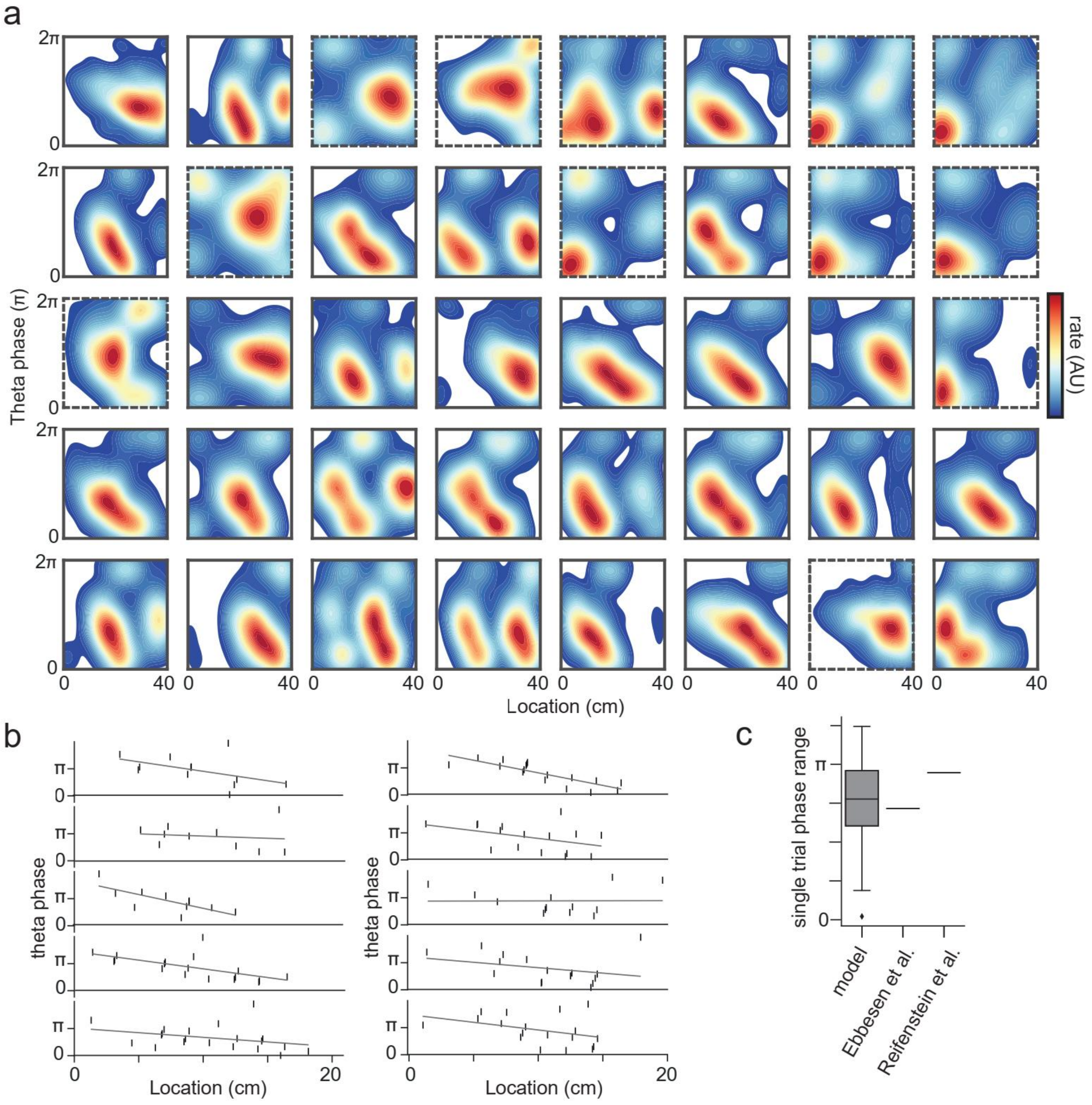

Supplementary Figure 2. Single trial phase precession. To allow comparison to identified EC stellate cells[4,5], we computed single trial phase precession for indicual grid cells of our model. **a** 40 random examples of phase precession plots of individual grid cells for a specific trajectory. For each cell, data are averaged over 20 Poisson seeds. Note, that even though every cell is defined as a perfectly phase precessing grid cell, many instances don't reflect the typical phase precession pattern (dashed frames). The reason is that for any physiologically plausible population of grid cells with diverse grid spacings, orientations and phase-offsets[3], a random trajectory will entail numerous cases where the center of a grid field is not traversed (compare Reifenstein et al., 2016[5] Fig. 1B). **b** Ten examples of linear fits to estimate phase range based on single trials (Poisson seeds), analogous to Ebbesen et al. (2016)[4] or Reifenstein et al. (2016)[5]. Fits were made to single field traversals (20 cm corresponds to 1s) **c** Estimates of single trial phase range for our model (n=10 grid traversals) and the literature (as data on the distribution was unavailable we plot only the reported medians). Note that we excluded cases that would lead to an artifactual depression of the estimated phase range (dashed frames in **a**).

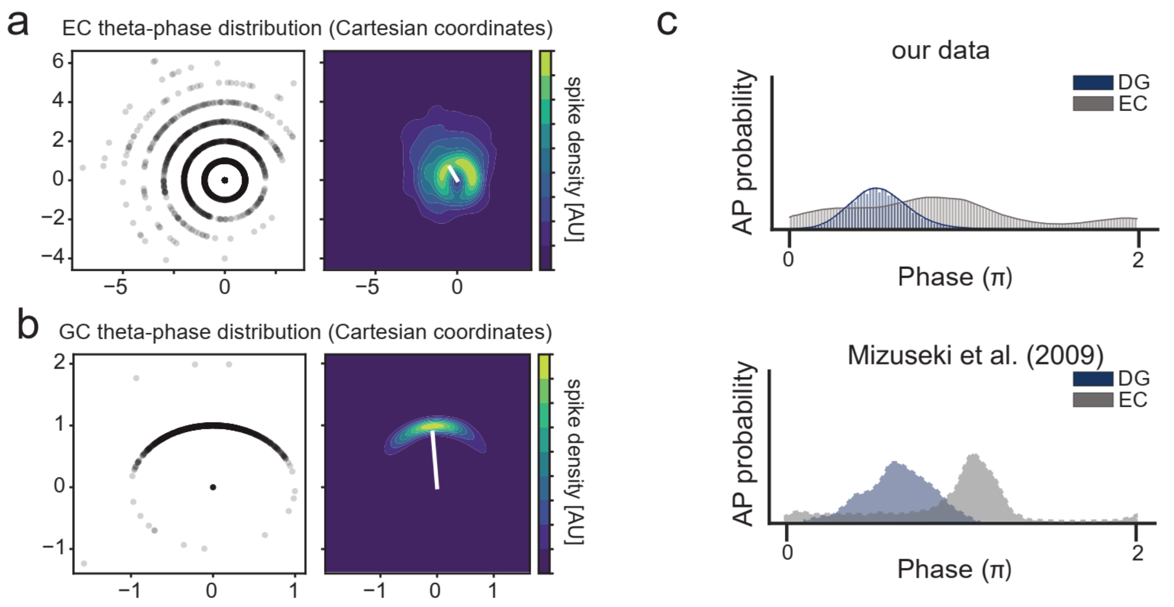

Supplementary Figure 3. Neuronal code in Cartesian space (or theta-space). The position of each bin in Cartesian space, where the angle of a data-point reflects the mean theta phase and the distance from origin (or vector length) reflects the mean rate (spikes per cycle). **a** Theta-phase distribution in EC. On the left is a scatter plot and on the right is a density plot with the resulting mean vector as a white line. Inactive bins are at [0,0] and they are omitted from the density plot for visual contrast (the density at [0,0] is several orders of magnitude higher than elsewhere). **b** Same as a but for DG granule cells. As expected from the narrow phase distribution and sparsity, the granule cell code is restricted to a small part of Cartesian space. The data is from a single grid seeds of a single Poisson seed at a single trajectory. **c** Comparison of our data with available *in vivo* data (Mizuseki et al., 2009; data was traced from Fig. 8, black dashed lines)[6]. To the best of our knowledge this is the only study allowing to directly compare theta-modulation between EC and DG. Note that both our model and *in vivo* data show a highly skewed distribution, where a mid-theta peak is preceded by what might be called an early theta plateau, where the DG activity peak 'precedes' the EC peak. Note that subsequent research suggests that the peak in the Mizuseki data is likely inflated by EC pyramidal cells[7,8], which do not project to DG and were not presently modeled.

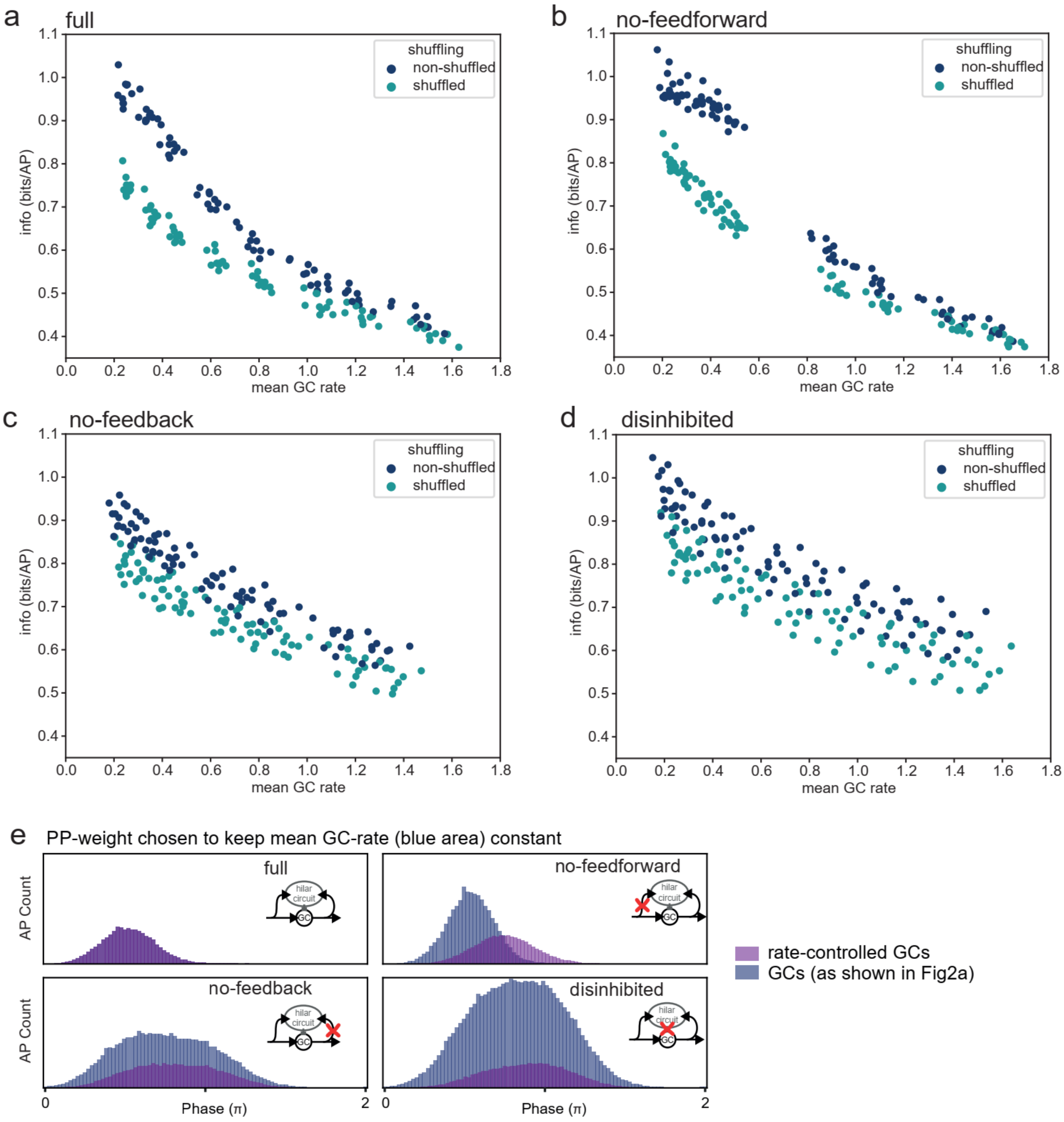

Supplementary Figure 4. Relation between GC sparsity and phase-to-rate coding. Perforant path strengths (synaptic weights) were systematically varied to produce a range of mean GC firing rates. This allowed us to pick and compare networks with 'controlled' GC sparsity (between 0.2 and 0.3 Hz). **a-d** mean spatial information per spike for each network for both non-shuffled and phase-shuffled data (each data point represents a grid seed). **e** theta-phase distributions for GCs when GC sparsity is controlled.

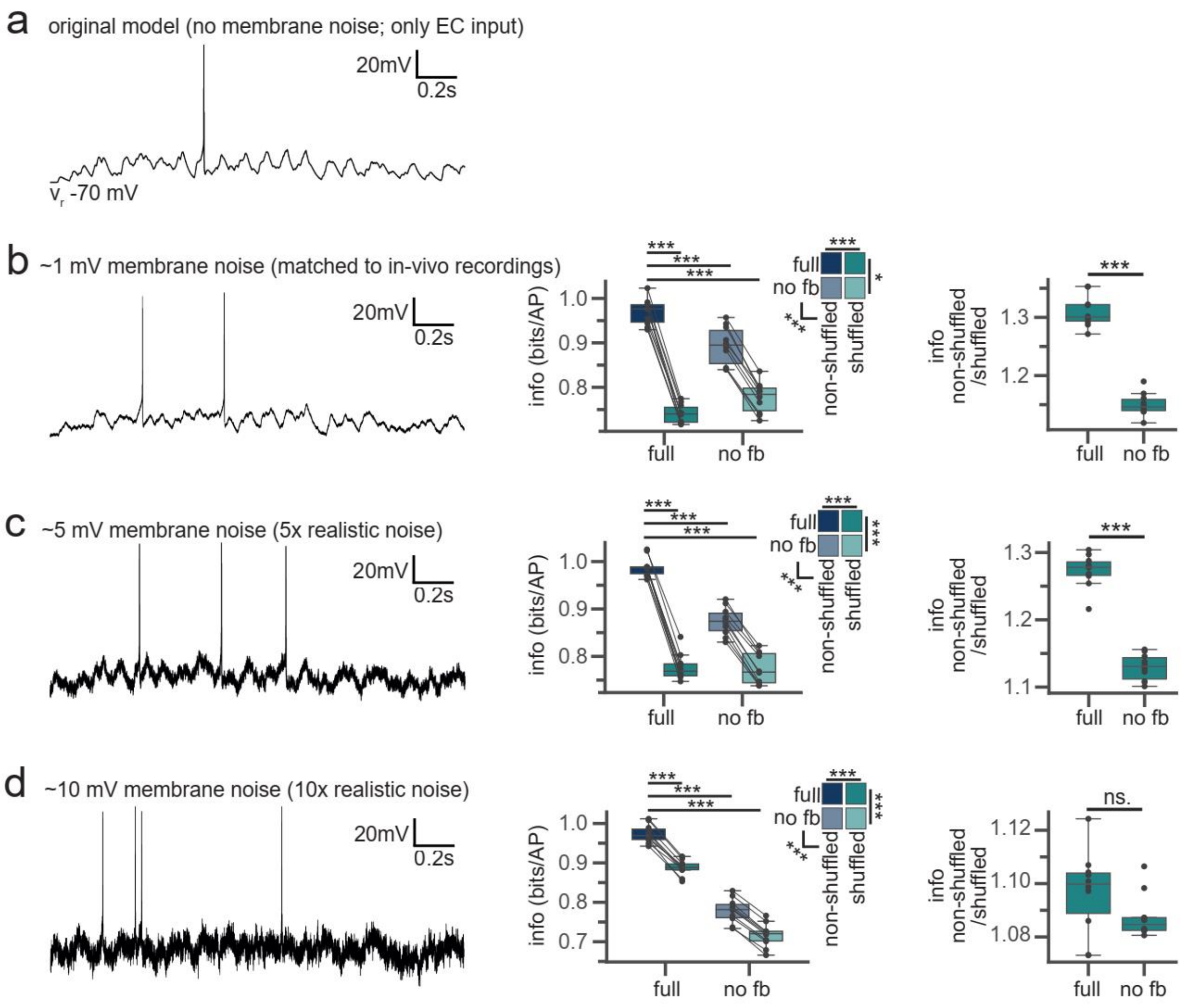

Supplementary Figure 5. Robustness of phase-to-rate recoding to GC membrane noise. Noise was simulated with a current injection at the soma of each granule cell. The noise current was drawn from a normal distribution of mean 0 and different standard deviations (sd) in **b**, **c** & **d** (all panels show the same cell on the left). **a** GC membrane voltage without noise. **b** Membrane noise (sd = 50pA) was chosen such that voltage traces matched in house *in vivo* recordings (also see [9]). On the right, results of spatial information analysis with GC rate-controlled data. **c,d** same as (b) but with ~5 and 10x realistic noise levels (sd=0.25 and 0.5nA, note that due to the low-pass filtering property of the membrane, voltage deflections are smaller than would be expected for constant current injections of the same amplitude). The significant phase-to-rate translation is robust to sd = 0.25 nA and lower membrane noise. At sd = 0.5 nA membrane noise the feedback inhibitory effect on shuffling is no longer significant. Two-way repeated measures ANOVA with Dunnett's post-test (middle) or paired t-test (right). n=10 grid seeds for all groups. * indicates $p<0.05$, *** $p<0.001$. Source data are provided in Source Data.xlsx. Full statistics are shown in supplementary tables 15-17.

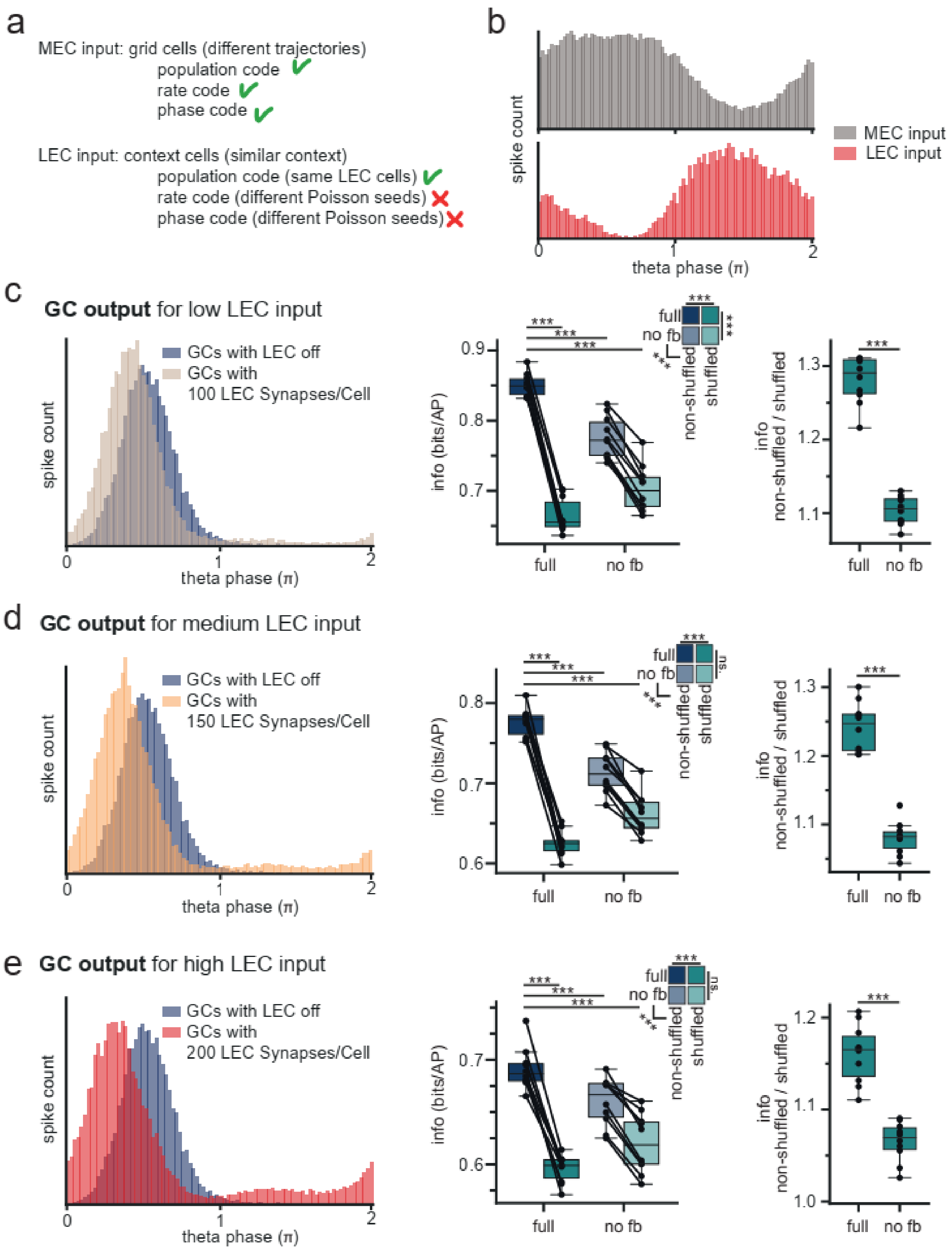

Supplementary Figure 6. Robustness of phase-to-rate recoding given counter-cyclical LEC input. LEC inputs were modeled as non-homogeneous Poisson spiking with countercyclical modulation and no spatial information. **a** LEC input properties: To model a coincident 'similar' contextual input from LEC, we assumed the exact same subpopulation of LEC cells were active, but contained no further rate or phase information (spike-trains were instantiated with different Poisson seeds). **b** Density distribution of action potentials for the MEC grid cell input used throughout the main part of the study (left) and the counter cyclical LEC input (right). All histograms of the figure contain data from a single grid seed with 20 Poisson seeds of a single trajectory. **c** On the left is the spike

count of granule cell spikes with the LEC input being off compared to 20 LEC cells, each contacting 100 randomly chosen granule cells. LEC synapses are located at the distal dendrite of the granule cells (425 µm from the soma). The LEC input makes more granule cells fire earlier and also causes more late firing. On the right are the results of the spatial information analysis. **d** & **e** show the same as **c** but with more LEC synapses. **e** shows that phase-to-rate recoding occurs even when the LEC synapses cause a strong shift in granule cell distribution. Two-way repeated measures ANOVA with Dunnett's post-test (middle) or paired t-test (right). n=10 grid seeds for all groups. *** indicates p<0.001. Source data are provided in Source Data.xlsx. Full statistics are shown in supplementary tables 18-20.

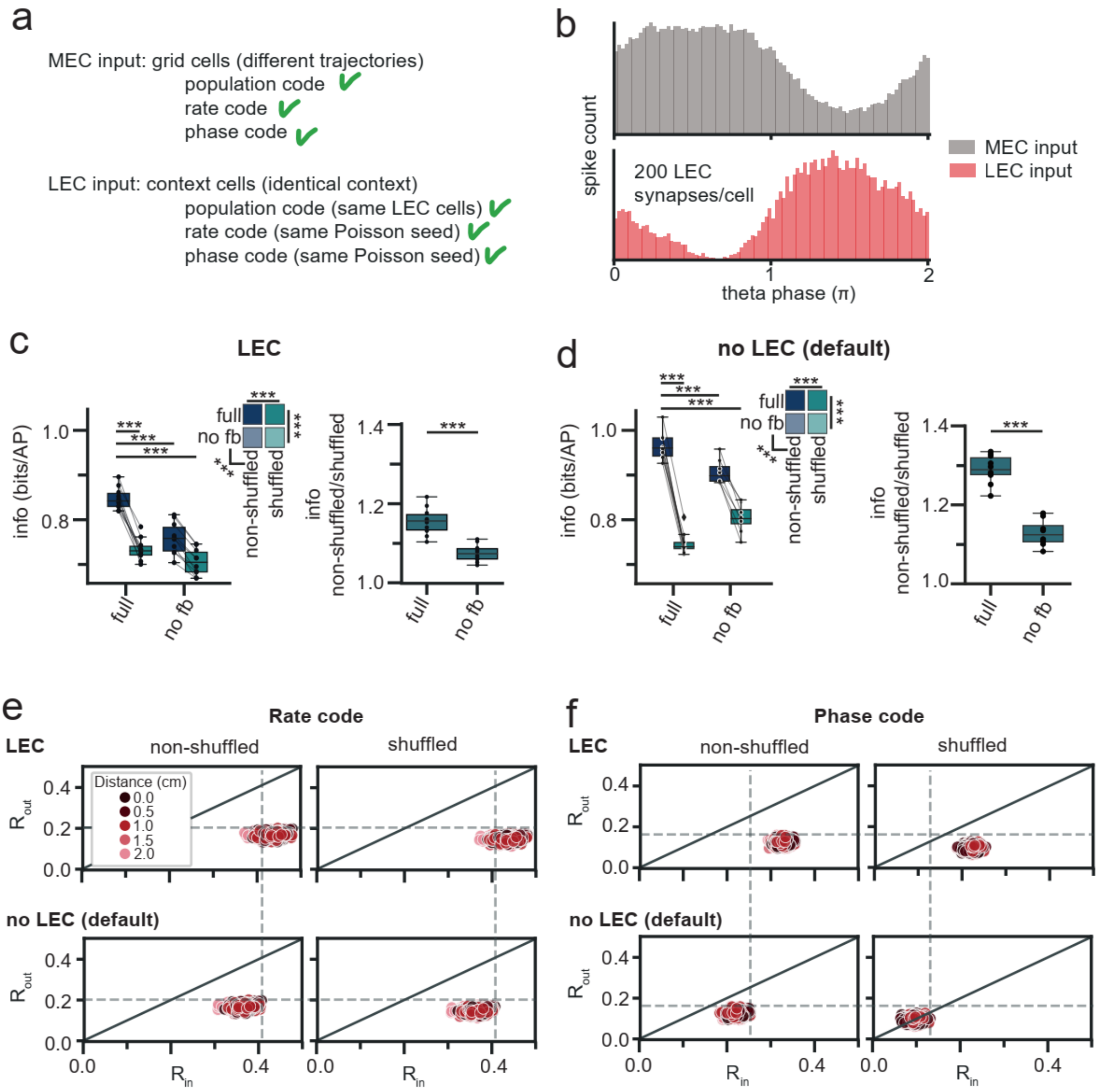

Supplementary Figure 7. Effect of simultaneous temporal codes from LEC. LEC inputs were again modeled as non-homogeneous Poisson spiking with countercyclical modulation (with 200 synapses per LEC cell), but this time the LEC-Poisson seed was held constant, meaning the exact same LEC spike-train is repeated. **a** This models the addition of a contextual LEC input with high (maximal) spatial and temporal information. **b** Illustration of the countercyclical theta modulation. **c** Skaggs spatial (rate) information in the dentate as in Supplementary Fig. 6 c-e **d** Same as c, but without the LEC input (same data as in Fig. 2i). **e** Rate code correlation plots of inputs (R_{in}) versus outputs (R_{out}) for a range of highly similar MEC patterns (Distances = 0 to 2 cm). The dashed grey lines indicate the maximal correlations for the non-shuffled, no LEC data. Notice that adding an 'identical' LEC input

increases input similarity (as expected) and still leads to robust pattern separation (maximal output similarity remains ≤0.2). **f** Same as e but for phase codes. Two-way repeated measures ANOVA with Dunnett's post-test (left) or paired t-test (right). n=10 grid seeds for all groups. *** indicates p<0.001. Source data are provided in Source Data.xlsx. Full statistics are shown in supplementary tables 21,22.

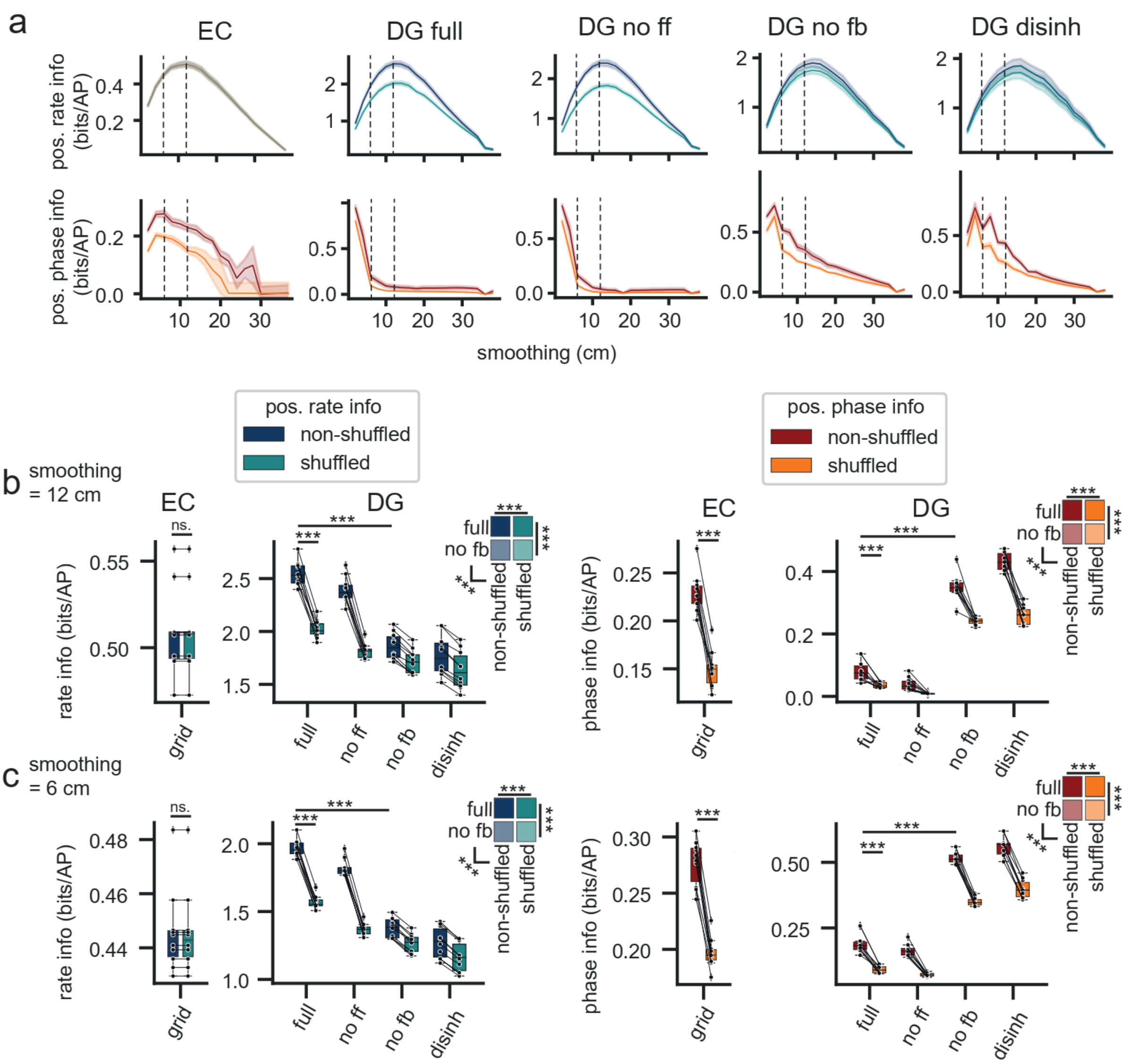

Supplementary Figure 8. Positional information analysis. Positional information[10] contained in rate and phase codes was computed according to Tingley & Buzsaki (2018)[11]. Axis labels are as in figs 1-6. As described previously[11], we found positional information to be highly dependent on the applied smoothing window. **a** Systematic analysis of positional rate (top) and phase (bottom) information for increasing smoothing windows. Note the general pattern consistent with previous analysis, whereby removing the feedback circuit (no fb) decreases the distance between the non-shuffled and shuffled curves for the rate code, but increases it for the phase code. **b** Quantification of positional rate (left) or phase (right) information for a smoothing window of 12 cm. **c** Quantification of positional rate (left) or phase (right) information for a smoothing window of 6 cm. Two-way repeated measures ANOVA with Dunnett's post-test (middle). n=10 grid seeds for all groups. *** indicates p<0.001. Note that not all significances are shown in the figure to avoid clutter. Source data are provided in Source Data.xlsx. Full statistics are shown in supplementary tables 23-27.

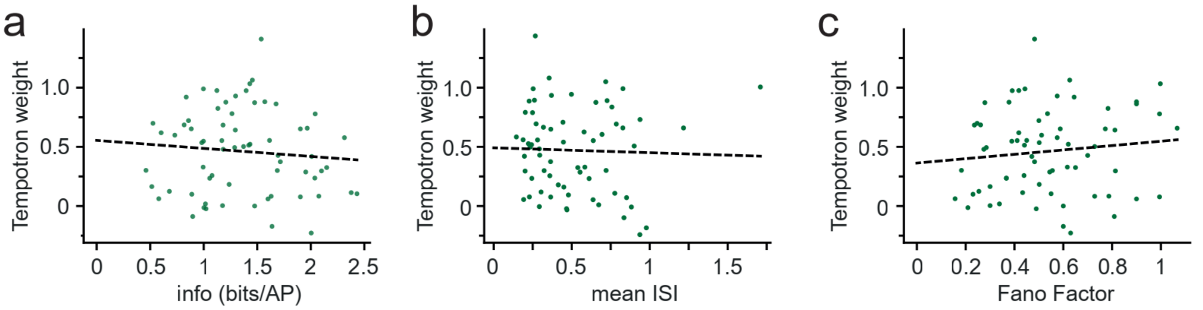

Supplementary Figure 9. Relation between GC spike train characteristics and relative tempotron weight. The spiking data from 200 randomly chosen GCs was used to train a tempotron for 200 epochs. The scatter plots show the trained tempotron weight of each GC that has more than 8 spikes across its 20 Poisson seeds (the criterion to compute Skaggs spatial information, see methods). **a** shows the trained tempotron weights against the Skaggs information measure. **b** shows the trained tempotron weights against the mean inter-spike interval (ISI). **c** shows the trained tempotron weights against the Fano factor. The statistical test of the linear regression's slope was non-significant (p>0.05) for all parameters.

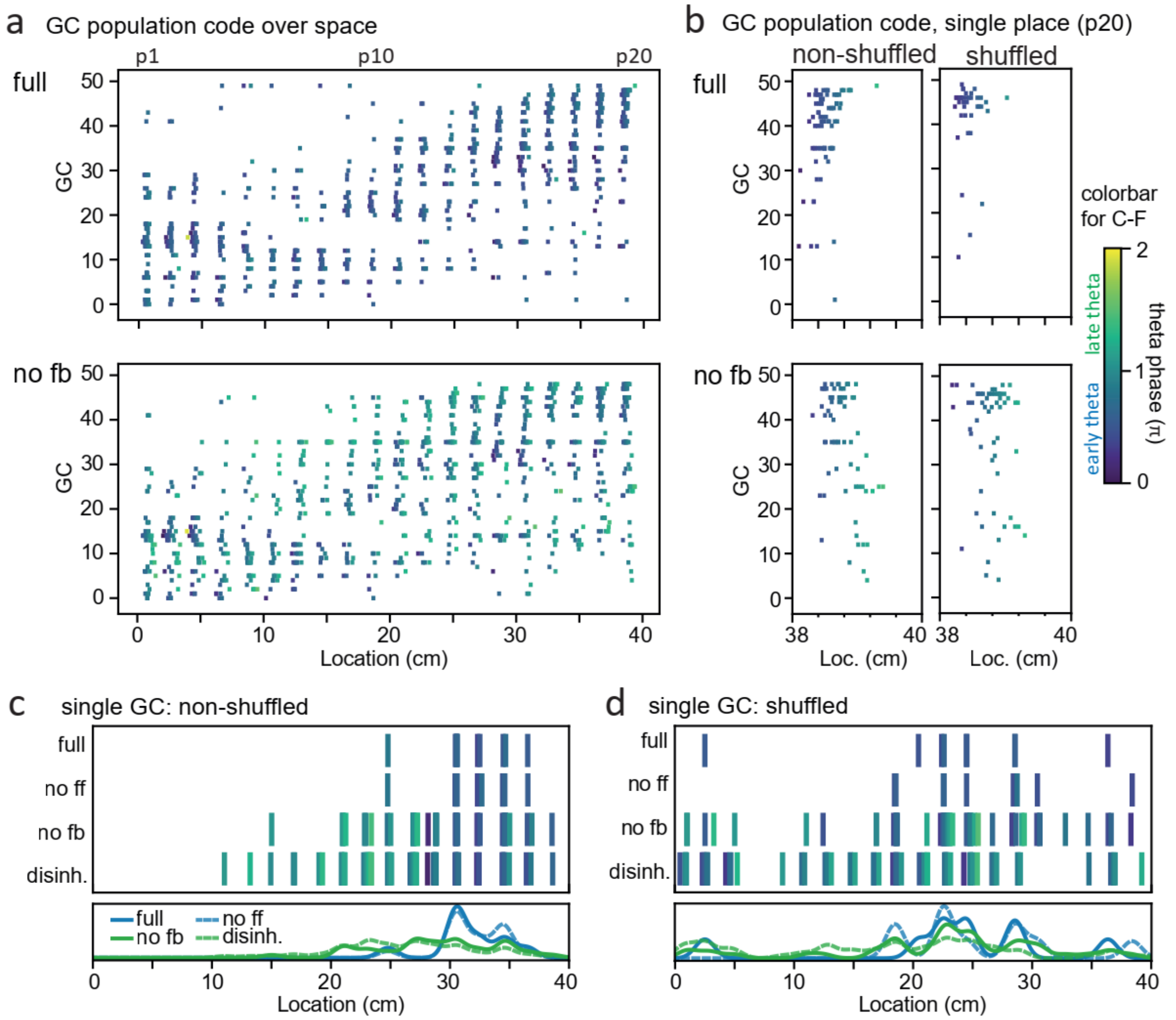

Supplementary Figure 10. Phase-to-rate recoding does not depend on changes in sparsity. a Same as Figure 5c but for GC rate-controlled data. **b** Same as Figure 5d but for GC rate-controlled data. **c** Individual GC with the highest difference in spatial information between full and no fb networks for non-shuffled data **d** GC selected as in c, but within the EC phase-shuffled data, to make sure that the observed pattern is not due to cell selection. Notice again the decreased spatial selectivity in non-shuffled vs shuffled, given equal sparsity (within network conditions), and the clear role of the inhibition of spatially selective late-theta spikes.

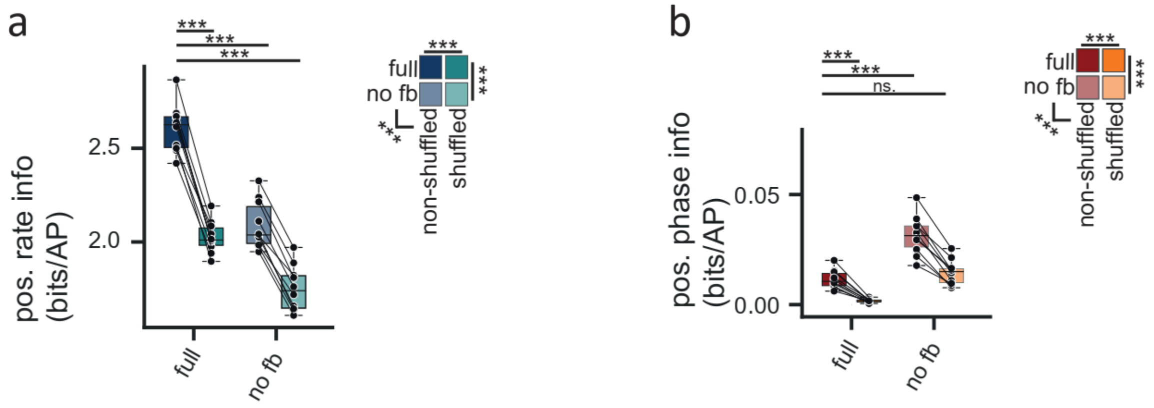

Supplementary Figure 11. Phase-to-rate recoding within only early theta. Positional information analysis[10,11] of rate and phase codes was performed for only early theta (i.e. ignoring all spikes occurring at phases > π). This ensures that information increases are not driven by the spatially selective inhibition of spikes in late theta. **a** Effect of removing feedback inhibition (no fb) on positional rate information. **b** Effect of removing feedback inhibition (no fb) on positional phase information. Two-way repeated measures ANOVA with Dunnett's post-test (middle). n=10 grid seeds for all groups. *** indicates p<0.001. Source data are provided in Source Data.xlsx. Full statistics are shown in supplementary tables 28,29.

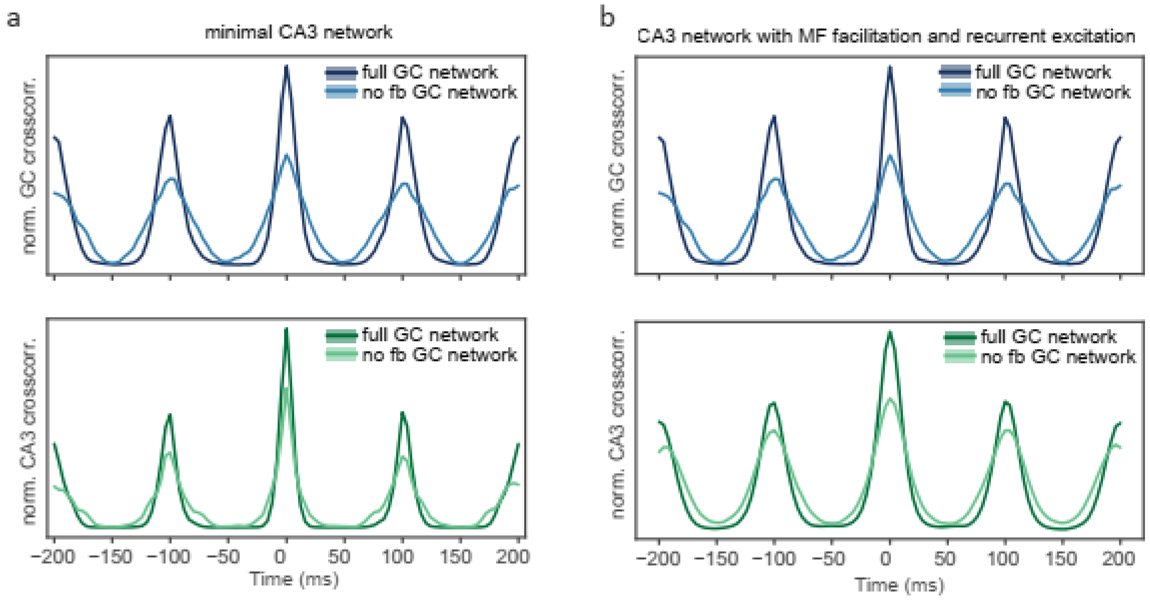

Supplementary Figure 12. Population cross-correlation in DG and CA3. The normalized population cross-correlogram was computed as the mean of pair-wise cross-correlograms between all cell-pairs in a population, (4 ms bins), normalized to the grand average across bins. **a** Population cross-correlograms of the minimal CA3 network for GCs (blue) and CA3 pyramidal cells (green) for the 'full' or rate-adjusted 'no feedback' (no fb) networks. **b** Population cross-correlograms of the extended CA3 network for GCs (blue) and CA3 pyramidal cells (green) for the 'full' or rate-adjusted 'no feedback' (no fb) networks.

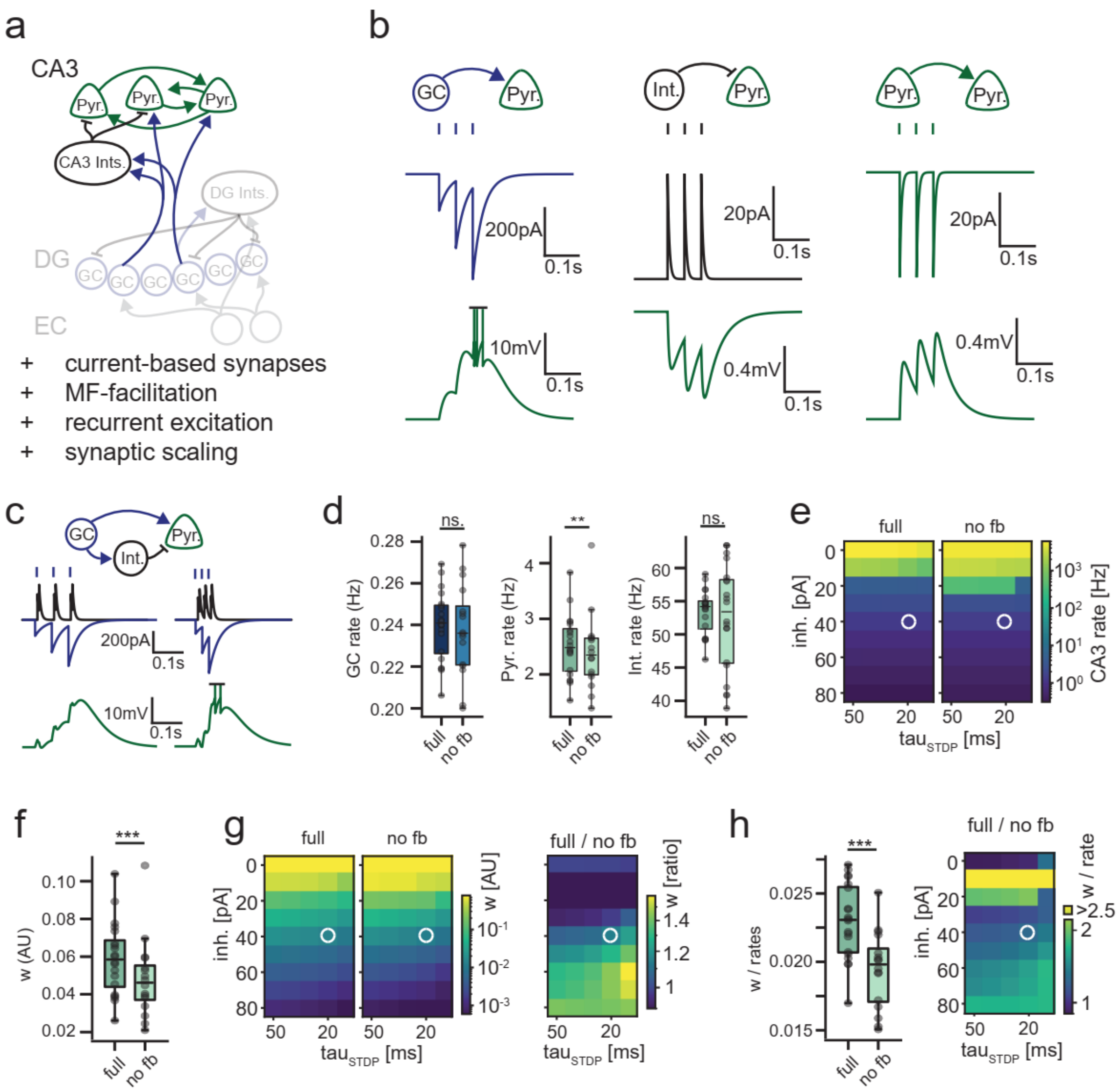

Supplementary Figure 13. Extended CA3 model. a Schematic of the CA3 model plus the added complexities of the extended model. **b** Examples of the modelled monosynaptic currents and voltages based on a brief 20Hz stimulus of three pulses (matched to Toth et al., 2000[12]). Truncated AP's in the voltage trace were added for illustrative purposes. Note the pronounced facilitation at the granule cell (GC) to CA3 Pyramidal (Pyr.) synapse. **c** Illustration of a combined excitatory/inhibitory input to a pyramidal cell, when accounting for approximate cell stoichiometry and mean firing rates (which lead to many IPSCs for any EPSC). Note that in this case a 20 Hz input does not suffice to fire the pyramidal cell, but a 50 Hz input does. **d** Mean firing rates of the extended model for 40 pA IPSC amplitude and 20 ms STDP timescale (white circles in e,g,h). **e** Dependence of mean CA3 pyramidal firing rate for different inhibitory strengths and STDP timescales. The empirically plausible range of activity for CA3 is 0.3 to 5Hz[13,14], i.e. the blue area in the plot. Note that, where this firing rate is exceeded (green and yellow area), the networks exhibits runaway excitation with implausibly high firing rates (≥200Hz). **f** Mean weight increases for 40 pA IPSC amplitude and 20 ms STDP timescale. **g** Dependence of mean weight increases for different inhibition strengths and STDP timescales, as well as the ratio between full and no feedback network (right). **h** Mean weight increase when weights are first normalized to mean rates (panel e). Note that values >2 (~0 to 20pA inhibition) fall into the range of runaway excitation and are arguably not meaningful. Source data are provided in Source Data.xlsx. Full statistics are shown in supplementary tables 30,31.

Supplementary Tables (Statistics)

**Fig. 1 k, l**	Mean deltaR, *rate-code*, non-shuffled	Mean deltaR, *Phase-code,* non-shuffled
Sample size	10	10
Minimum	0,09178004	0,0332957
25% Percentile	0,1090441	0,04299277
Median	0,1216964	0,04715239
75% Percentile	0,1263486	0,05652955
Maximum	0,1305200	0,06074433
Mean	0,1177772	0,04869256
Std. Deviation	0,01206557	0,008732343
Std. Error of Mean	0,003815467	0,002761409
Lower 95% CI of mean	0,1091459	0,04244577
Upper 95% CI of mean	0,1264084	0,05493936
D'Agostino & Pearson omnibus normality test		
K2	4,157759	0,3515745
P value	0,1251	0,8388
Passed normality test (alpha=0.05)?	Yes	Yes
P value summary	ns	ns
One sample t test		
Theoretical mean	0,0	0,0
Actual mean	0,1177772	0,04869256
Discrepancy	-0,1177772	-0,04869256
95% CI of discrepancy	0,1091466 to 0,1264077	0,04244625 to 0,05493887
t, df	t=30,86835 df=9	t=17,63323 df=9
P value (two tailed)	< 0,0001	< 0,0001
Significant (alpha=0.05)?	Yes	Yes
Sum	1,177772	0,4869256

Supplementary Table 1. Statistics for Fig. 1 k,l - Mean deltaR, non-shuffled

**Fig. 2c**	GC firing rates				
**Two-way RM ANOVA**	Matching: Both factors				
Alpha	0,05				
Source of Variation	% of total variation	P value	P value summary	Significant?	
network	98,50	< 0,0001	****	Yes	
shuffling	0,3529	< 0,0001	****	Yes	
Interaction: network x shuffling	0,2034	< 0,0001	****	Yes	
Interaction: network x Subjects	0,3411				
Interaction: shuffling x Subjects	0,004254				
Subjects	0,6011				
ANOVA table	SS	DF	MS	F (DFn, DFd)	P value
network	16,59	3	5,529	F (3, 27) = 2599	P < 0,0001
shuffling	0,05942	1	0,05942	F (1, 9) = 746,6	P < 0,0001
Interaction: network x shuffling	0,03425	3	0,01142	F (3, 27) = 1438	P < 0,0001
Interaction: network x Subjects	0,05745	27	0,002128		
Interaction: shuffling x Subjects	0,0007163	9	7,959e-005		
Subjects	0,1012	9	0,01125		
Residual	0,0002144	27	7,941e-006		

**Post-hoc tests**								
Number of families	1							
Number of comparisons per family	4							
Alpha	0,05							
Bonferroni's multiple comparisons	Mean Diff,	95% CI of diff,	Significant?	Summary	Adjusted P Value			
non-shuffled - shuffled								
granule full	-0,01306	-0,01644 to -0,009691	Yes	****	< 0,0001			
granule no-feedforward	-0,03143	-0,03481 to -0,02806	Yes	****	< 0,0001			
granule no-feedback	-0,05129	-0,05466 to -0,04791	Yes	****	< 0,0001			
granule disinhibited	-0,1223	-0,1256 to -0,1189	Yes	****	< 0,0001			
Test details	Mean 1	Mean 2	Mean Diff,	SE of diff,	N1	N2	t	DF
non-shuffled - shuffled								
granule full	0,2407	0,2538	-0,01306	0,001260	10	10	10,37	27
granule no-feedforward	0,4116	0,4430	-0,03143	0,001260	10	10	24,94	27
granule no-feedback	0,7299	0,7812	-0,05129	0,001260	10	10	40,70	27
granule disinhibited	1,379	1,502	-0,1223	0,001260	10	10	97,01	27

Supplementary Table 2. Statistics for Fig. 2c – GC firing rates

**Fig. 2 e, f**	Mean deltaR, *rate-code*, shuffled	Mean deltaR, *phase-code,* shuffled
Number of values	10	10
Minimum	0,1059820	4,030000e-005
25% Percentile	0,1363758	0,0005860
Median	0,1395690	0,0020330
75% Percentile	0,1439393	0,0037495
Maximum	0,1493470	0,0049970
Mean	0,1373747	0,00223653
Std. Deviation	0,01178902	0,001713373
Std. Error of Mean	0,003728014	0,0005418162
Lower 95% CI of mean	0,1289413	0,001010846
Upper 95% CI of mean	0,1458081	0,003462214
D'Agostino & Pearson omnibus normality test		
K2	19,40891	1,306609
P value	< 0,0001	0,5203
Passed normality test (alpha=0.05)?	No	Yes
P value summary	****	ns
One sample t test		
Theoretical mean	0,0	0,0
Actual mean	0,1373747	0,00223653
Discrepancy	-0,1373747	-0,00223653
95% CI of discrepancy	0,1289419 to 0,1458075	0,001010942 to 0,003462118
t, df	t=36,84930 df=9	t=4,127839 df=9
P value (two tailed)	< 0,0001	0,0026
Significant (alpha=0.05)?	Yes	Yes
Sum	1,373747	0,0223653

Supplementary Table 3. Statistics for Fig. 2e,f – Mean deltaR, shuffled

Fig. 2g	Mean deltaR				
**Two-way RM ANOVA**	Matching: Both factors				
Alpha	0,05				
Source of Variation	% of total variation	P value	P value summary	Significant?	
network	75,64	< 0,0001	****	Yes	
shuffling	3,942	< 0,0001	****	Yes	
Interaction: network x shuffling	1,855	< 0,0001	****	Yes	
Interaction: network x Subjects	3,334				
Interaction: shuffling x Subjects	0,5721				
Subjects	14,36				
ANOVA table	SS	DF	MS	F (DFn, DFd)	P value
network	0,06048	3	0,02016	F (3, 27) = 204,2	P < 0,0001
shuffling	0,003152	1	0,003152	F (1, 9) = 62,02	P < 0,0001
Interaction: network x shuffling	0,001483	3	0,0004944	F (3, 27) = 56,74	P < 0,0001
Interaction: network x Subjects	0,002666	27	9,873e-005		
Interaction: shuffling x Subjects	0,0004574	9	5,083e-005		
Subjects	0,01148	9	0,001276		
Residual	0,0002353	27	8,714e-006		

**Post-hoc tests**								
Number of families	1							
Number of comparisons per family	4							
Alpha	0,05							
Bonferroni's multiple comparisons test	Mean Diff,	95% CI of diff,	Significant?	Summary	Adjusted P Value			
non-shuffled - shuffled								
granule full	-0,01960	-0,02313 to -0,01606	Yes	****	< 0,0001			
granule no-feedforward	-0,02257	-0,02610 to -0,01903	Yes	****	< 0,0001			
granule no-feedback	-0,004832	-0,008365 to -0,001299	Yes	**	0,0043			
granule disinhibited	-0,003220	-0,006753 to 0,0003131	No	ns	0,0863			
Test details	Mean 1	Mean 2	Mean Diff,	SE of diff,	N1	N2	t	DF
non-shuffled - shuffled								
granule full	0,1178	0,1374	-0,01960	0,001320	10	10	14,85	27
granule no-feedforward	0,1021	0,1247	-0,02257	0,001320	10	10	17,09	27
granule no-feedback	0,08177	0,08660	-0,004832	0,001320	10	10	3,660	27
granule disinhibited	0,05456	0,05778	-0,003220	0,001320	10	10	2,439	27tt

Supplementary Table 4. Statistics for Fig. 2g - Mean deltaR

Fig. 2h	Skaggs-nonadjusted				
**Two-way RM ANOVA**	Matching: Both factors				
Alpha	0,05				
Source of Variation	% of total variation	P value	P value summary	Significant?	
network	55,32	< 0,0001	****	Yes	
shuffling	32,68	< 0,0001	****	Yes	
Interaction: network x shuffling	7,447	< 0,0001	****	Yes	
Interaction: network x Subjects	0,9881				
Interaction: shuffling x Subjects	0,1732				
Subjects	3,204				
ANOVA table	SS	DF	MS	F (DFn, DFd)	P value
network	0,8208	3	0,2736	F (3, 27) = 503,8	P < 0,0001
shuffling	0,4849	1	0,4849	F (1, 9) = 1698	P < 0,0001
Interaction: network x shuffling	0,1105	3	0,03683	F (3, 27) = 348,8	P < 0,0001
Interaction: network x Subjects	0,01466	27	0,0005430		
Interaction: shuffling x Subjects	0,002571	9	0,0002856		
Subjects	0,04754	9	0,005282		
Residual	0,002851	27	0,0001056		

.

**Post-hoc tests**								
Number of families	1							
Number of comparisons per family	4							
Alpha	0,05							
Bonferroni's multiple comparisons test	Mean Diff,	95% CI of diff,	Significant?	Summary	Adjusted P Value			
non-shuffled - shuffled								
granule full	0,2168	0,2045 to 0,2291	Yes	****	< 0,0001			
granule no-feedforward	0,2422	0,2299 to 0,2545	Yes	****	< 0,0001			
granule no-feedback	0,08091	0,06861 to 0,09320	Yes	****	< 0,0001			
granule disinhibited	0,08295	0,07065 to 0,09525	Yes	****	< 0,0001			
Test details	Mean 1	Mean 2	Mean Diff,	SE of diff,	N1	N2	t	DF
non-shuffled - shuffled								
granule full	0,9659	0,7491	0,2168	0,004595	10	10	47,19	27
granule no-feedforward	0,9314	0,6892	0,2422	0,004595	10	10	52,70	27
granule no-feedback	0,7234	0,6425	0,08091	0,004595	10	10	17,61	27
granule disinhibited	0,6434	0,5605	0,08295	0,004595	10	10	18,05	27

Supplementary Table 5. Statistics for Fig. 2h - Skaggs-nonadjusted

**Fig. 2i**	Skaggs-adjusted				
**Two-way RM ANOVA**	Matching: Across row				
Alpha	0,05				
Source of Variation	% of total variation	P value	P value summary	Significant?	
Interaction	6,043185	< 0,0001	****	Yes	
network	1,691157	0,1341	ns	No	
shuffling	76,60512	< 0,0001	****	Yes	
Subjects (matching)	9,937244	< 0,0001	****	Yes	
ANOVA table	SS	DF	MS	F (DFn, DFd)	P value
Interaction	0,03732219	3	0,01244073	F (3, 35) = 55,3065	P < 0,0001
network	0,01044444	3	0,00348148	F (3, 35) = 1,98548	P = 0,1341
shuffling	0,4731066	1	0,4731066	F (1, 35) = 2103,25	P < 0,0001
Subjects (matching)	0,06137157	35	0,001753473	F (35, 35) = 7,79525	P < 0,0001
Residual	0,007872941	35	0,0002249412		
Number of missing values	0				

**Post-hoc tests**								
Number of families	1							
Number of comparisons per family	4							
Alpha	0,05							
Bonferroni's multiple comparisons test	Mean Diff,	95% CI of diff,	Significant?	Summary	Adjusted P Value			
non-shuffled - shuffled								
granule full	0,2168359	0,19917 to 0,23450	Yes	****	< 0,0001			
granule no-feedforward	0,1812252	0,16438 to 0,19807	Yes	****	< 0,0001			
granule no-feedback	0,1029354	0,083188 to 0,12268	Yes	****	< 0,0001			
granule disinhibited	0,1263302	0,10867 to 0,14399	Yes	****	< 0,0001			
Test details	Mean 1	Mean 2	Mean Diff,	SE of diff,	N1	N2	t	DF
non-shuffled - shuffled								
granule full	0,9659343	0,7490984	0,2168359	0,006707327	10	10	32,33	35
granule no-feedforward	0,9650850	0,7838598	0,1812252	0,006395185	11	11	28,34	35
granule no-feedback	0,9078136	0,8048782	0,1029354	0,00749902	8	8	13,73	35
granule disinhibited	0,9473096	0,8209794	0,1263302	0,006707327	10	10	18,83	35

Supplementary Table 6. Statistics for Fig. 2i - Skaggs-adjusted

**Fig. 2j**	Skaggs-adjusted non-shuffled to shuffled ratio				
**One-way ANOVA**					
F	58,80				
P value	< 0,0001				
P value summary	****				
Are differences among means statistically significant? (P < 0.05)	Yes				
R square	0,8344				
Brown-Forsythe test					
F (DFn, DFd)	1,314 (3, 35)				
P value	0,2852				
P value summary	ns				
Significantly different standard deviations? (P < 0.05)	No				
Bartlett's test					
Bartlett's statistic (corrected)	3,738				
P value	0,2912				
P value summary	ns				
Significantly different standard deviations? (P < 0.05)	No				
ANOVA table	SS	DF	MS	F (DFn, DFd)	P value
Treatment (between columns)	0,1525	3	0,05084	F (3, 35) = 58,80	P < 0,0001
Residual (within columns)	0,03026	35	0,0008647		
Total	0,1828	38			
Data summary					
Number of treatments (columns)	4				
Number of values (total)	39				

**Post-hoc tests**								
Number of comparisons per family	3							
Alpha	0,05							
Dunnett's multiple comparisons test	Mean Diff,	95% CI of diff,	Significant?	Summary	Adjusted P Value			
full vs. noFF	0,05833	0,02679 to 0,08988	Yes	***	0,0002			
full vs. noFB	0,1612	0,1270 to 0,1955	Yes	****	< 0,0001			
full vs. disinh	0,1360	0,1037 to 0,1682	Yes	****	< 0,0001			
Test details	Mean 1	Mean 2	Mean Diff,	SE of diff,	n1	n2	q	DF
full vs. noFF	1,290	1,232	0,05833	0,01285	10	11	4,540	35
full vs. noFB	1,290	1,129	0,1612	0,01395	10	8	11,56	35
full vs. disinh	1,290	1,154	0,1360	0,01315	10	10	10,34	35

Supplementary Table 7. Statistics for Fig. 2j - Skaggs-adjusted non-shuffled to shuffled ratio

Fig. 3h	Rate perceptron				
**Repeated measures ANOVA**					
Assume sphericity?	No				
F	202,0				
P value	< 0,0001				
P value summary	****				
Statistically significant (P < 0.05)?	Yes				
Geisser-Greenhouse's epsilon	0,6402				
R square	0,9573				
Was the matching effective?					
F	3,116				
P value	0,0106				
P value summary	*				
Is there significant matching (P < 0.05)?	Yes				
R square	0,04242				
ANOVA table	SS	DF	MS	F (DFn, DFd)	P value
Treatment (between columns)	0,2270	3	0,07565	F (1,920, 17,28) = 202,0	P < 0,0001
Individual (between rows)	0,01050	9	0,001167	F (9, 27) = 3,116	P = 0,0106
Residual (random)	0,01011	27	0,0003745		
Total	0,2476	39			
Data summary					
Number of treatments (columns)	4				
Number of subjects (rows)	10				

**Post-hoc tests (two-tailed)**								
Number of families	1							
Number of comparisons per family	3							
Alpha	0,05							
Holm-Sidak's multiple comparisons test	Mean Diff,	Significant?	Summary	Adjusted P Value				
full granule vs. no-feedforward granule	-0,003628	No	ns	0,6921				
full granule vs. no-feedback granule	0,1369	Yes	****	< 0,0001				
full granule vs. disinhibited granule	0,1591	Yes	****	< 0,0001				
Test details	Mean 1	Mean 2	Mean Diff,	SE of diff,	n1	n2	t	DF
full granule vs. no-feedforward granule	1,171	1,174	-0,003628	0,008871	10	10	0,4090	9
full granule vs. no-feedback granule	1,171	1,034	0,1369	0,008604	10	10	15,92	9
full granule vs. disinhibited granule	1,171	1,012	0,1591	0,008703	10	10	18,28	9

Supplementary Table 8. Statistics for Fig. 3h - Rate perceptron

**Fig. 3i**	Phase perceptron				
**Repeated measures ANOVA**					
Assume sphericity?	No				
F	227,4				
P value	< 0,0001				
P value summary	****				
Statistically significant (P < 0.05)?	Yes				
Geisser-Greenhouse's epsilon	0,7075				
R square	0,9619				
Was the matching effective?					
F	4,343				
P value	0,0014				
P value summary	**				
Is there significant matching (P < 0.05)?	Yes				
R square	0,05223				
ANOVA table	SS	DF	MS	F (DFn, DFd)	P value
Treatment (between columns)	0,3704	3	0,1235	F (2,122, 19,10) = 227,4	P < 0,0001
Individual (between rows)	0,02122	9	0,002358	F (9, 27) = 4,343	P = 0,0014
Residual (random)	0,01466	27	0,0005430		
Total	0,4063	39			
Data summary					
Number of treatments (columns)	4				
Number of subjects (rows)	10				

**Post-hoc tests**								
Number of families	1							
Number of comparisons per family	3							
Alpha	0,05							
Holm-Sidak's multiple comparisons test	Mean Diff,	Significant?	Summary	Adjusted P Value				
full granule vs. no-feedforward granule	0,02252	Yes	*	0,0393				
full granule vs. no-feedback granule	-0,1793	Yes	****	< 0,0001				
full granule vs. disinhibited granule	-0,1817	Yes	****	< 0,0001				
Test details	Mean 1	Mean 2	Mean Diff,	SE of diff,	n1	n2	t	DF
full granule vs. no-feedforward granule	1,210	1,187	0,02252	0,009348	10	10	2,409	9
full granule vs. no-feedback granule	1,210	1,389	-0,1793	0,01187	10	10	15,10	9
full granule vs. disinhibited granule	1,210	1,392	-0,1817	0,009863	10	10	18,43	9

Supplementary Table 9. Statistics for Fig. 3i - Phase perceptron

**Fig. 3j**	GC-rate controlled rate perceptron				
**Repeated measures ANOVA**					
Assume sphericity?	No				
F	215,3				
P value	< 0,0001				
P value summary	****				
Statistically significant (P < 0.05)?	Yes				
Geisser-Greenhouse's epsilon	0,6307				
R square	0,9599				
Was the matching effective?					
F	3,579				
P value	0,0048				
P value summary	**				
Is there significant matching (P < 0.05)?	Yes				
R square	0,04568				
ANOVA table	SS	DF	MS	F (DFn, DFd)	P value
Treatment (between columns)	0,3116	3	0,1039	F (1,892, 17,03) = 215,3	P < 0,0001
Individual (between rows)	0,01554	9	0,001726	F (9, 27) = 3,579	P = 0,0048
Residual (random)	0,01302	27	0,0004824		
Total	0,3401	39			
Data summary					
Number of treatments (columns)	4				
Number of subjects (rows)	10				

**Post-hoc tests**								
Number of families	1							
Number of comparisons per family	3							
Alpha	0,05							
Holm-Sidak's multiple comparisons test	Mean Diff,	Significant?	Summary	Adjusted P Value				
full granule vs. no-feedforward granule	0,06955	Yes	****	< 0,0001				
full granule vs. no-feedback granule	0,1806	Yes	****	< 0,0001				
full granule vs. disinhibited granule	0,2227	Yes	****	< 0,0001				
Test details	Mean 1	Mean 2	Mean Diff,	SE of diff,	n1	n2	t	DF
full granule vs. no-feedforward granule	1,175	1,105	0,06955	0,008068	10	10	8,621	9
full granule vs. no-feedback granule	1,175	0,9943	0,1806	0,01304	10	10	13,85	9
full granule vs. disinhibited granule	1,175	0,9521	0,2227	0,01198	10	10	18,59	9

Supplementary Table 10. Statistics for Fig. 3j - GC-rate controlled rate perceptron

**Fig. 3k**	GC-rate controlled phase perceptron				
Two-way RM ANOVA summary					
Assume sphericity?	No				
F	33,84				
P value	< 0,0001				
P value summary	****				
Statistically significant (P < 0.05)?	Yes				
Geisser-Greenhouse's epsilon	0,4689				
R square	0,7899				
Was the matching effective?					
F	4,440				
P value	0,0012				
P value summary	**				
Is there significant matching (P < 0.05)?	Yes				
R square	0,2372				
ANOVA table	SS	DF	MS	F (DFn, DFd)	P value
Treatment (between columns)	0,05109	3	0,01703	F (1,407, 12,66) = 33,84	P < 0,0001
Individual (between rows)	0,02011	9	0,002234	F (9, 27) = 4,440	P = 0,0012
Residual (random)	0,01359	27	0,0005032		
Total	0,08479	39			
Data summary					
Number of treatments (columns)	4				
Number of subjects (rows)	10				

**Post-hoc tests**								
Number of families	1							
Number of comparisons per family	3							
Alpha	0,05							
Holm-Sidak's multiple comparisons test	Mean Diff,	Significant?	Summary	Adjusted P Value				
full granule vs. no-feedforward granule	0,04532	Yes	**	0,0011				
full granule vs. no-feedback granule	-0,00423	No	ns	0,7733				
full granule vs. disinhibited granule	0,08298	Yes	***	0,0006				
Test details	Mean 1	Mean 2	Mean Diff,	SE of diff,	n1	n2	t	DF
full granule vs. no-feedforward granule	1,215	1,170	0,04532	0,00873	10	10	5,191	9
full granule vs. no-feedback granule	1,215	1,219	-0,00423	0,01426	10	10	0,297	9
full granule vs. disinhibited granule	1,215	1,132	0,08298	0,01375	10	10	6,034	9

Supplementary Table 11. Statistics for Fig. 3k - GC-rate controlled phase perceptron

**Fig. 4c**	Tempotron				
Two-way RM ANOVA	Matching: Both factors				
Assume sphericity	No				
Alpha	0,05				
Source of Variation	% of total variation	P value	P value summary	Significant?	
shuffling	2,208382	0,0391	*	Yes	
network	5,815663	0,0041	**	Yes	
Interaction: shuffling x network	1,912656	0,0623	ns	No	
Interaction: shuffling x Subjects	13,19924				
Interaction: network x Subjects	16,67945				
Subjects	45,97417				
ANOVA table	SS	DF	MS	F (DFn, DFd)	P value
shuffling	6,828181e-005	1	6,828181e-005	F (1, 28) = 4,684715	P = 0,0391
network	0,0001798167	1	0,0001798167	F (1, 28) = 9,762824	P = 0,0041
Interaction: shuffling x network	5,913814e-005	1	5,913814e-005	F (1, 28) = 3,768667	P = 0,0623
Interaction: shuffling x Subjects	0,0004081125	28	1,457545e-005		
Interaction: network x Subjects	0,0005157184	28	1,841851e-005		
Subjects	0,001421493	28	5,076761e-005		
Residual	0,0004393776	28	1,569206e-005		

**Post tests**								
Number of families	1							
Number of comparisons per family	3							
Alpha	0,05							
Dunnet's multiple comparisons test	Mean Diff,	95% CI of diff,	Significant?	Summary	Adjusted P Value			
non-shuffled:full vs. non-shuffled:no fb	0,00392	0,00141 to 0,00643	Yes	**	0,0017			
non-shuffled:full vs. shuffled:full	0,00296	0,00077 to 0,00516	Yes	**	0,0064			
non-shuffled:full vs. shuffled:no fb	0,00402	0,00058 to 0,00746	Yes	*	0,0191			
Test details	Mean 1	Mean 2	Mean Diff,	SE of diff,	N1	N2	t	DF
non-shuffled:full vs. non-shuffled:no fb	0,01973	0,01581	0,003918	0,001011	29	29	3,877	28
non-shuffled:full vs. shuffled:full	0,01973	0,01677	0,002962	0,000884	29	29	3,350	28
non-shuffled:full vs. shuffled:no fb	0,01973	0,01571	0,004025	0,001385	29	29	2,905	28

Supplementary Table 12. Statistics for Fig. 4c – Tempotron

**Fig. 6b**	GC rates
Column B	noFB
vs.	vs,
Column A	full
Unpaired t test with Welch's correction	
P value	0,8840
P value summary	ns
Significantly different? (P < 0.05)	No
One- or two-tailed P value?	Two-tailed
Welch-corrected t, df	t=0,1467097 df=43,21926
How big is the difference?	
Mean ± SEM of column A	0,2396665 ± 0,003016508 N=30
Mean ± SEM of column B	0,2404056 ± 0,004035261 N=23
Difference between means	0,0007391409 ± 0,00503812
95% confidence interval	-0,009419663 to 0,01089794
R square	0,0004977645
F test to compare variances	
F,DFn, Dfd	1,371958, 22, 29
P value	0,4205
P value summary	ns
Significantly different? (P < 0.05)	No

**Fig. 6c**	CA3 Pyr. rates
Column B	noFB
vs.	vs,
Column A	full
Unpaired t test with Welch's correction	
P value	<0,0001
P value summary	****
Significantly different (P < 0.05)?	Yes
One- or two-tailed P value?	Two-tailed
Welch-corrected t, df	t=14,75525, df=44,78073
How big is the difference?	
Mean ± SEM of column A	3,182425 ± 0,0910157 N=30
Mean ± SEM of column B	1,635005 ± 0,0520997 N=23
Difference between means (B - A) ± SEM	-1,547420 ± 0,1048725
95% confidence interval	-1,758672 to -1,336167
R squared (eta squared)	0,8294055
F test to compare variances	
F, DFn, Dfd	3,980667, 29, 22
P value	0,0014
P value summary	**
Significantly different (P < 0.05)?	Yes

**Fig. 6c**	CA3 Int. rates
Column B	noFB
vs.	vs,
Column A	full
Unpaired t test with Welch's correction	
P value	< 0,0001
P value summary	****
Significantly different? (P < 0.05)	Yes
One- or two-tailed P value?	Two-tailed
Welch-corrected t, df	t=15,87634 df=24,78663
How big is the difference?	
Mean ± SEM of column A	43,74065 ± 0,1379504 N=30
Mean ± SEM of column B	55,08281 ± 0,7009612 N=24
Difference between means	11,34216 ± 0,7144067
95% confidence interval	9,870157 to 12,81416
R square	0,9104673
F test to compare variances	
F,DFn, Dfd	20,65534, 23, 29
P value	< 0,0001
P value summary	****
Significantly different? (P < 0.05)	Yes

Supplementary Table 13. Statistics for Fig. 6b,c – Firing rates

Fig. 6f	Mean weight-increase, symmetric STDP
Column B	noFB
vs.	vs,
Column A	full
Unpaired t test with Welch's correction	
P value	< 0,0001
P value summary	****
Significantly different? (P < 0.05)	Yes
One- or two-tailed P value?	Two-tailed
Welch-corrected t, df	t=11,67248 df=36,85646
How big is the difference?	
Mean ± SEM of column A	0,2403164 ± 0,01451026 N=30
Mean ± SEM of column B	0,05929604 ± 0,005473519 N=23
Difference between means	-0,1810203 ± 0,01550829
95% confidence interval	-0,2124472 to -0,1495934
R square	0,7870840
F test to compare variances	
F,DFn, Dfd	9,166656, 29, 22
P value	< 0,0001
P value summary	****
Significantly different? (P < 0.05)	Yes

Fig. 6h	Mean weight-increase/rate, symmetric STDP
Column B	noFB
vs.	vs,
Column A	full
Unpaired t test with Welch's correction	
P value	< 0,0001
P value summary	****
Significantly different? (P < 0.05)	Yes
One- or two-tailed P value?	Two-tailed
Welch-corrected t, df	t=12,93080 df=50,49588
How big is the difference?	
Mean ± SEM of column A	0,07366017 ± 0,00234817 N=30
Mean ± SEM of column B	0,03503939 ± 0,001845709 N=23
Difference between means	-0,03862077 ± 0,002986728
95% confidence interval	-0,04461839 to -0,03262316
R square	0,7680498
F test to compare variances	
F,DFn, Dfd	2,111185, 29, 22
P value	0,0747
P value summary	ns
Significantly different? (P < 0.05)	No

Fig. 6j	Mean weight-increase, assymmetric STDP
Column B	noFB
vs.	vs,
Column A	full
Unpaired t test with Welch's correction	
P value	< 0,0001
P value summary	****
Significantly different? (P < 0.05)	Yes
One- or two-tailed P value?	Two-tailed
Welch-corrected t, df	t=13,79463 df=36,77894
How big is the difference?	
Mean ± SEM of column A	0,0765540 ± 0,003896508 N=30
Mean ± SEM of column B	0,01914422 ± 0,001461976 N=23
Difference between means	-0,05740978 ± 0,004161749
95% confidence interval	-0,0658439 to -0,04897566
R square	0,8380287
F test to compare variances	
F,DFn, Dfd	9,265404, 29, 22
P value	< 0,0001
P value summary	****
Significantly different? (P < 0.05)	Yes

Fig. 6l	Mean weight-increase/rate, assymmetric STDP
Column B	noFB
vs.	vs,
Column A	full
Unpaired t test with Welch's correction	
P value	< 0,0001
P value summary	****
Significantly different? (P < 0.05)	Yes
One- or two-tailed P value?	Two-tailed
Welch-corrected t, df	t=16,31636 df=50,93923
How big is the difference?	
Mean ± SEM of column A	0,0240000 ± 0,0005407123 N=30
Mean ± SEM of column B	0,01212022 ± 0,0004875918 N=23
Difference between means	-0,01187978 ± 0,0007280903
95% confidence interval	-0,01334154 to -0,01041802
R square	0,8393909
F test to compare variances	
F,DFn, Dfd	1,604033, 29, 22
P value	0,2567
P value summary	ns
Significantly different? (P < 0.05)	No

Supplementary Table 14. Statistics for Fig. 6f-I – Mean synaptic weight increases in CA3

Suppl. Fig. 5b	1mV Noise Robustness				
**Two-way RM ANOVA**	Matching: Both factors				
Assume sphericity	No				
Alpha	0,05				
Source of Variation	% of total variation	P value	P value summary	Significant?	GG epsilon
Shuffling	80,17	<0,0001	****	Yes	1,000
Network	1,014	0,0353	*	Yes	1,000
shuffling x network	8,439	<0,0001	****	Yes	1,000
grid_seed x shuffling	0,4373				
grid_seed x network	1,491				
grid_seed	8,128				
ANOVA table	SS	DF	MS	F (DFn, DFd)	P value
shuffling	0,2997	1	0,2997	F (1,000, 9,000) = 1650	P<0,0001
network	0,003792	1	0,003792	F (1,000, 9,000) = 6,123	P=0,0353
shuffling x network	0,03155	1	0,03155	F (1,000, 9,000) = 237,2	P<0,0001
grid_seed x shuffling	0,001635	9	0,0001816		
grid_seed x network	0,005574	9	0,0006194		
grid_seed	0,03038	9	0,003376		
Residual	0,001197	9	0,0001330		
.					

**Post-hoc test**								
Number of families	1							
Number of comparisons per	3							
Alpha	0,05							
Dunnett's multiple comparisons	Mean Diff,	95,00% CI of diff,	Below	Summary	Adjusted P			
non-shuffled:full granule vs.	0,07564	0,04840 to 0,1029	Yes	****	<0,0001			
non-shuffled:full granule vs.	0,2293	0,2124 to 0,2462	Yes	****	<0,0001			
non-shuffled:full granule vs.	0,1926	0,1695 to 0,2157	Yes	****	<0,0001			
Test details	Mean 1	Mean 2	Mean Diff,	SE of diff,	N1	N2	q	DF
non-shuffled:full granule vs.	0,9696	0,8940	0,07564	0,009689	10	10	7,806	9,000
non-shuffled:full granule vs.	0,9696	0,7403	0,2293	0,006019	10	10	38,09	9,000
non-shuffled:full granule vs.	0,9696	0,7770	0,1926	0,008229	10	10	23,40	9,000
**Paired t test**	Non-shuffled/shuffled							
no fb vs. full								
P value	<0,0001	Mean of differences	-0,1594	Correlation (r)	0,01160			
P value summary	****	SD of differences	0,03316	one tailed P	0,4873			
Significance (P < 0.05)?	Yes	SEM of differences	0,01049	P value	ns			
One- or two-tailed P value?	Two-tailed	R squared	0,9625	Sign. Pairing?	No			
t, df	t=15,20, df=9							
Number of pairs	10							

Supplementary Table 15. Statistics for Supplementary Fig. 5b – Baseline membrane noise injection

Suppl. Fig. 5c	5mV Noise Robustness				
**Two-way RM ANOVA**	Matching: Both factors				
Assume sphericity	No				
Alpha	0,05				
Source of Variation	% of total variation	P value	P value summary	Significant?	GG epsilon
shuffling	71,97	<0,0001	****	Yes	1,000
network	10,30	<0,0001	****	Yes	1,000
shuffling x network	9,282	<0,0001	****	Yes	1,000
grid_seed x shuffling	0,3109				
grid_seed x network	1,463				
grid_seed	6,495				
ANOVA table	SS	DF	MS	F (DFn, DFd)	P value
shuffling	0,2424	1	0,2424	F (1,000, 9,000) = 2084	P<0,0001
network	0,03468	1	0,03468	F (1,000, 9,000) = 63,34	P<0,0001
shuffling x network	0,03126	1	0,03126	F (1,000, 9,000) = 469,7	P<0,0001
grid_seed x shuffling	0,001047	9	0,0001163		
grid_seed x network	0,004928	9	0,0005476		
grid_seed	0,02188	9	0,002431		
Residual	0,0005990	9	6,655e-005		

.

**Post-hoc test**								
Number of families	1							
Number of comparisons per	3							
Alpha	0,05							
Dunnett's multiple comparisons	Mean Diff,	95,00% CI of diff,	Below	Summary	Adjusted P			
non-shuffled:full granule vs.	0,1148	0,09330 to 0,1363	Yes	****	<0,0001			
non-shuffled:full granule vs.	0,2116	0,1990 to 0,2242	Yes	****	<0,0001			
non-shuffled:full granule vs.	0,2146	0,1903 to 0,2388	Yes	****	<0,0001			
Test details	Mean 1	Mean 2	Mean Diff,	SE of diff,	N1	N2	q	DF
non-shuffled:full granule vs.	0,9888	0,8739	0,1148	0,007647	10	10	15,01	9,000
non-shuffled:full granule vs.	0,9888	0,7771	0,2116	0,004488	10	10	47,15	9,000
non-shuffled:full granule vs.	0,9888	0,7742	0,2146	0,008625	10	10	24,88	9,000

**Paired t test**	Non-shuffled/shuffled						
no fb vs. full							
P value	<0,0001	Mean of differences	-0,1436	Correlation (r)	0,3884		
P value summary	****	SD of differences	0,02491	one tailed P	0,1337		
Significance (P < 0.05)?	Yes	SEM of differences	0,007876	P value	ns		
One- or two-tailed P value?	Two-tailed	R squared	0,9736	Sign. Pairing?	No		
t, df	t=18,23, df=9						
Number of pairs	10						

Supplementary Table 16. Statistics for Supplementary Fig. 5c – 5mV standard deviation membrane noise injection

Suppl. Fig. 5d	10mV Noise Robustness				
**Two-way RM ANOVA**	Matching: Both factors				
Assume sphericity	No				
Alpha	0,05				
Source of Variation	% of total variation	P value	P value summary	Significant?	GG epsilon
shuffling	13,24	<0,0001	****	Yes	1,000
network	80,30	<0,0001	****	Yes	1,000
shuffling x network	0,3220	0,0003	***	Yes	1,000
grid_seed x shuffling	0,1342				
grid_seed x network	1,949				
grid_seed	3,962				
ANOVA table	SS	DF	MS	F (DFn, DFd)	P value
shuffling	0,05515	1	0,05515	F (1,000, 9,000) = 887,7	P<0,0001
network	0,3346	1	0,3346	F (1,000, 9,000) = 370,8	P<0,0001
shuffling x network	0,001342	1	0,001342	F (1,000, 9,000) = 31,20	P=0,0003
grid_seed x shuffling	0,0005592	9	6,213e-005		
grid_seed x network	0,008123	9	0,0009026		
grid_seed	0,01651	9	0,001834		
Residual	0,0003870	9	4,300e-005		
.					

**Post-hoc test**								
Number of families	1							
Number of comparisons per	3							
Alpha	0,05							
Dunnett's multiple comparisons	Mean Diff,	95,00% CI of diff,	Below	Summary	Adjusted P			
non-shuffled:full granule vs.	0,1945	0,1633 to 0,2257	Yes	****	<0,0001			
non-shuffled:full granule vs.	0,08585	0,07383 to 0,09786	Yes	****	<0,0001			
non-shuffled:full granule vs.	0,2572	0,2274 to 0,2870	Yes	****	<0,0001			
Test details	Mean 1	Mean 2	Mean Diff,	SE of diff,	N1	N2	q	DF
non-shuffled:full granule vs.	0,9734	0,7789	0,1945	0,01109	10	10	17,55	9,000
non-shuffled:full granule vs.	0,9734	0,8875	0,08585	0,004272	10	10	20,09	9,000
non-shuffled:full granule vs.	0,9734	0,7162	0,2572	0,01058	10	10	24,30	9,000

**Paired t test**	Non-shuffled/shuffled					
no fb vs. full						
P value	0,0570	Mean of differences	-0,009130	Correlation (r)	0,5338	
P value summary	ns	SD of differences	0,01324	one tailed P	0,0560	
Significance (P < 0.05)?	No	SEM of differences	0,004185	P value	ns	
One- or two-tailed P value?	Two-tailed	R squared	0,3459	Sign. Pairing?	No	
t, df	t=2,181, df=9					
Number of pairs	10					

Supplementary Table 17. Statistics for Supplementary Fig. 5d – 10mV standard deviation membrane noise injection

**Suppl. Fig. 6c**	Similar LEC 100 Syn. Robustness				
**Two-way RM ANOVA**	Matching: Both factors				
Assume sphericity	No				
Alpha	0,05				
Source of Variation	% of total variation	P value	P value summary	Significant?	Geisser-
shuffling	73,34	<0,0001	****	Yes	1,000
network	1,443	0,0464	*	Yes	1,000
shuffling x network	14,08	<0,0001	****	Yes	1,000
grid_seed x shuffling	0,6107				
grid_seed x network	2,437				
grid_seed	7,895				
ANOVA table	SS	DF	MS	F (DFn, DFd)	P value
shuffling	0,1678	1	0,1678	F (1,000, 9,000) = 1081	P<0,0001
network	0,003300	1	0,003300	F (1,000, 9,000) = 5,328	P=0,0464
shuffling x network	0,03222	1	0,03222	F (1,000, 9,000) = 650,2	P<0,0001
grid_seed x shuffling	0,001397	9	0,0001552		
grid_seed x network	0,005575	9	0,0006194		
grid_seed	0,01806	9	0,002007		
Residual	0,0004459	9	4,955e-005		
.					

**Post-hoc test**								
Number of families	1							
Number of comparisons per	3							
Alpha	0,05							
Dunnett's multiple comparisons	Mean Diff,	95,00% CI of diff,	Below	Summary	Adjusted P			
ns:full vs. ns:no-feedback	0,07493	0,04913 to 0,1007	Yes	****	<0,0001			
ns:full vs. s:full	0,1863	0,1719 to 0,2007	Yes	****	<0,0001			
ns:full vs. s:no-feedback	0,1477	0,1201 to 0,1753	Yes	****	<0,0001			
Test details	Mean 1	Mean 2	Mean Diff,	SE of diff,	N1	N2	q	DF
ns:full vs. ns:no-feedback	0,8508	0,7759	0,07493	0,009174	10	10	8,167	9,000
ns:full vs. s:full	0,8508	0,6645	0,1863	0,005119	10	10	36,39	9,000
ns:full vs. s:no-feedback	0,8508	0,7031	0,1477	0,009805	10	10	15,06	9,000

**Paired t test**	Non-shuffled/shuffled						
no fb vs. full							
P value	<0,0001	Mean of differences	-0,1773	Correlation (r)	0,6602		
P value summary	****	SD of differences	0,02413	one tailed P	0,0189		
Significance (P < 0.05)?	Yes	SEM of differences	0,007631	P value	*		
One- or two-tailed P value?	Two-tailed	R squared	0,9836	Sign. Pairing?	Yes		
t, df	t=23,23, df=9						
Number of pairs	10						

Supplementary Table 18. Statistics for Supplementary Fig. 6c – 100 LEC synapses per cell

**Suppl. Fig. 6d**	Similar LEC 150 Syn. Robustness				
**Two-way RM ANOVA**	Matching: Both factors				
Assume sphericity	No				
Alpha	0,05				
Source of Variation	% of total variation	P value	P value summary	Significant?	Geisser-
shuffling	70,95	<0,0001	****	Yes	1,000
network	1,146	0,1005	ns	No	1,000
shuffling x network	16,46	<0,0001	****	Yes	1,000
grid_seed x shuffling	1,356				
grid_seed x network	3,081				
grid_seed	6,407				
ANOVA table	SS	DF	MS	F (DFn, DFd)	P value
shuffling	0,1039	1	0,1039	F (1,000, 9,000) = 471,0	P<0,0001
network	0,001678	1	0,001678	F (1,000, 9,000) = 3,349	P=0,1005
shuffling x network	0,02410	1	0,02410	F (1,000, 9,000) = 245,0	P<0,0001
grid_seed x shuffling	0,001985	9	0,0002205		
grid_seed x network	0,004511	9	0,0005012		
grid_seed	0,009380	9	0,001042		
Residual	0,0008850	9	9,834e-005		
.					

**Post-hoc test**								
Number of families	1							
Number of comparisons per	3							
Alpha	0,05							
Dunnett's multiple comparisons	Mean Diff,	95,00% CI of diff,	Below	Summary	Adjusted P			
ns:full vs. ns:no-feedback	0,06204	0,04105 to 0,08304	Yes	****	<0,0001			
ns:full vs. s:full	0,1510	0,1332 to 0,1688	Yes	****	<0,0001			
ns:full vs. s:no-feedback	0,1149	0,09158 to 0,1382	Yes	****	<0,0001			
Test details	Mean 1	Mean 2	Mean Diff,	SE of diff,	N1	N2	q	DF
ns:full vs. ns:no-feedback	0,7761	0,7140	0,06204	0,007466	10	10	8,309	9,000
ns:full vs. s:full	0,7761	0,6251	0,1510	0,006341	10	10	23,81	9,000
ns:full vs. s:no-feedback	0,7761	0,6612	0,1149	0,008283	10	10	13,87	9,000

**Paired t test**	Non-shuffled/shuffled						
no fb vs. full							
P value	<0,0001	Mean of differences	-0,1619	Correlation (r)	0,3769		
P value summary	****	SD of differences	0,03454	one tailed P	0,1415		
Significance (P < 0.05)?	Yes	SEM of differences	0,01092	P value	ns		
One- or two-tailed P value?	Two-tailed	R squared	0,9606	Sign. Pairing?	No		
t, df	t=14,82, df=9						
Number of pairs	10						

Supplementary Table 19. Statistics for Supplementary Fig. 6d – 150 LEC synapses per cell

Suppl. Fig. 6e	Similar LEC 200 Syn. Robustness				
**Two-way RM ANOVA**	Matching: Both factors				
Assume sphericity	No				
Alpha	0,05				
Source of Variation	% of total variation	P value	P value summary	Significant?	Geisser-
shuffling	64,96	<0,0001	****	Yes	1,000
network	0,1733	0,6607	ns	No	1,000
shuffling x network	10,82	<0,0001	****	Yes	1,000
grid_seed x shuffling	1,573				
grid_seed x network	7,571				
grid_seed	13,35				
ANOVA table	SS	DF	MS	F (DFn, DFd)	P value
shuffling	0,04605	1	0,04605	F (1,000, 9,000) = 371,5	P<0,0001
network	0,0001228	1	0,0001228	F (1,000, 9,000) = 0,2060	P=0,6607
shuffling x network	0,007669	1	0,007669	F (1,000, 9,000) = 62,58	P<0,0001
grid_seed x shuffling	0,001115	9	0,0001239		
grid_seed x network	0,005367	9	0,0005963		
grid_seed	0,009465	9	0,001052		
Residual	0,001103	9	0,0001225		

.

**Post-hoc test**								
Number of families	1							
Number of comparisons per	3							
Alpha	0,05							
Dunnett's multiple comparisons	Mean Diff,	95,00% CI of diff,	Below	Summary	Adjusted P			
ns:full vs. ns:no-feedback	0,03120	0,009205 to 0,05319	Yes	**	0,0082			
ns:full vs. s:full	0,09555	0,07926 to 0,1118	Yes	****	<0,0001			
ns:full vs. s:no-feedback	0,07136	0,04598 to 0,09675	Yes	****	<0,0001			
Test details	Mean 1	Mean 2	Mean Diff,	SE of diff,	N1	N2	q	DF
ns:full vs. ns:no-feedback	0,6914	0,6602	0,03120	0,007822	10	10	3,989	9,000
ns:full vs. s:full	0,6914	0,5959	0,09555	0,005794	10	10	16,49	9,000
ns:full vs. s:no-feedback	0,6914	0,6201	0,07136	0,009028	10	10	7,905	9,000

**Paired t test**	Non-shuffled/shuffled				
no fb vs. full					
P value	<0,0001	Mean of differences	-0,09520	Correlation (r)	0,01524
P value summary	****	SD of differences	0,03805	one tailed P	0,4833
Significance (P < 0.05)?	Yes	SEM of differences	0,01203	P value	ns
One- or two-tailed P value?	Two-tailed	R squared	0,8743	Sign. Pairing?	No
t, df	t=7,911, df=9				
Number of pairs	10				

Supplementary Table 20. Statistics for Supplementary Fig. 6e – 200 LEC synapses per cell

**Suppl. Fig. 7c**	Identical LEC 200 Syn. Robustness				
**Two-way RM ANOVA**	Matching: Both factors				
Assume sphericity	No				
Alpha	0,05				
Source of Variation	% of total variation	P value	P value summary	Significant?	Geisser-
shuffling	48,73	<0,0001	****	Yes	1,000
network	23,77	<0,0001	****	Yes	1,000
shuffling x network	6,378	<0,0001	****	Yes	1,000
grid_seed x shuffling	1,402				
grid_seed x network	3,057				
grid_seed	15,65				
ANOVA table	SS	DF	MS	F (DFn, DFd)	P value
shuffling	0,06968	1	0,06968	F (1,000, 9,000) = 312,8	P<0,0001
network	0,03398	1	0,03398	F (1,000, 9,000) = 69,96	P<0,0001
shuffling x network	0,009120	1	0,009120	F (1,000, 9,000) = 56,25	P<0,0001
grid_seed x shuffling	0,002005	9	0,0002227		
grid_seed x network	0,004371	9	0,0004857		
grid_seed	0,02237	9	0,002486		
Residual	0,001459	9	0,0001621		

.

**Post-hoc test**								
Number of families	1							
Number of comparisons per	3							
Alpha	0,05							
Dunnett's multiple comparisons	Mean Diff,	95,00% CI of diff,	Below	Summary	Adjusted P			
ns:full vs. ns:no-feedback	0,08849	0,06591 to 0,1111	Yes	****	<0,0001			
ns:full vs. s:full	0,1137	0,09303 to 0,1343	Yes	****	<0,0001			
ns:full vs. s:no-feedback	0,1418	0,1209 to 0,1626	Yes	****	<0,0001			
Test details	Mean 1	Mean 2	Mean Diff,	SE of diff,	N1	N2	q	DF
ns:full vs. ns:no-feedback	0,8470	0,7585	0,08849	0,008031	10	10	11,02	9,000
ns:full vs. s:full	0,8470	0,7333	0,1137	0,007341	10	10	15,49	9,000
ns:full vs. s:no-feedback	0,8470	0,7052	0,1418	0,007422	10	10	19,10	9,000

**Paired t test**	Non-shuffled/shuffled						
no fb vs. full							
P value	<0,0001	Mean of differences	-0,08012	Correlation (r)	0,1351		
P value summary	****	SD of differences	0,03824	one tailed P	0,3549		
Significance (P < 0.05)?	Yes	SEM of differences	0,01209	P value	ns		
One- or two-tailed P value?	Two-tailed	R squared	-0,1075 to -	Sign. Pairing?	No		
t, df	t=6,626, df=9		0,8299				
Number of pairs	10						

Supplementary Table 21. Statistics for Supplementary Fig. 7c – 200 LEC synapses per cell, identical input

**Suppl. Fig. 7d**	no LEC for comparison (note: fewer iterations were simulated than in the main fig. 2i)				
**Two-way RM ANOVA**	Matching: Both factors				
Assume sphericity	No				
Alpha	0,05				
Source of Variation	% of total variation	P value	P value summary	Significant?	Geisser-
shuffling	80,35	<0,0001	****	Yes	1,000
network	0,04378	0,7865	ns	No	1,000
shuffling x network	10,77	<0,0001	****	Yes	1,000
grid_seed x shuffling	0,1968				
grid_seed x network	3,869				
grid_seed	3,570				
ANOVA table	SS	DF	MS	F (DFn, DFd)	P value
shuffling	0,2110	1	0,2110	F (1,000, 7,000) = 2858	P<0,0001
network	0,0001149	1	0,0001149	F (1,000, 7,000) = 0,07921	P=0,7865
shuffling x network	0,02828	1	0,02828	F (1,000, 7,000) = 62,85	P<0,0001
grid_seed x shuffling	0,0005168	7	7,382e-005		
grid_seed x network	0,01016	7	0,001451		
grid_seed	0,009374	7	0,001339		
Residual	0,003149	7	0,0004499		
.					

**Post-hoc test**								
Number of families	1							
Number of comparisons per	3							
Alpha	0,05							
Dunnett's multiple comparisons	Mean Diff,	95,00% CI of diff,	Below	Summary	Adjusted P			
ns:full vs. ns:no-feedback	0,06324	0,01803 to 0,1085	Yes	*	0,0107			
ns:full vs. s:full	0,2218	0,2001 to 0,2435	Yes	****	<0,0001			
ns:full vs. s:no-feedback	0,1662	0,1256 to 0,2068	Yes	****	<0,0001			
Test details	Mean 1	Mean 2	Mean Diff,	SE of diff,	N1	N2	q	DF
ns:full vs. ns:no-feedback	0,9711	0,9078	0,06324	0,01522	8	8	4,155	7,000
ns:full vs. s:full	0,9711	0,7492	0,2218	0,007301	8	8	30,38	7,000
ns:full vs. s:no-feedback	0,9711	0,8049	0,1662	0,01366	8	8	12,16	7,000

**Paired t test**	Non-shuffled/shuffled						
no fb vs. full							
P value	<0,0001	Mean of differences	-0,1677	Correlation (r)	-0,7377		
P value summary	****	SD of differences	0,05954	one tailed P	0,0184		
Significance (P < 0.05)?	Yes	SEM of differences	0,02105	P value	*		
One- or two-tailed P value?	Two-tailed	R squared	0,9007	Sign. Pairing?	Yes		
t, df	t=7,968, df=7						
Number of pairs	8						

Supplementary Table 22. Statistics for Supplementary Fig. 7d – no LEC synapses (same as Fig.2i) for comparison

**Suppl. Fig. 8b rate**	Positional rate information analysis, 12cm smoothing				
**Two-way RM ANOVA**	Matching: Both factors				
Assume sphericity?	No				
Alpha	0,05				
Source of Variation	% of total variation	P value	P value summary	Significant?	Geisser-
network	10,09	0,0895	ns	No	0,3392
shuffling	27,15	<0,0001	****	Yes	1,000
network x shuffling	6,426	<0,0001	****	Yes	0,6283
Subject x network	25,26				
Subject x shuffling	1,291				
Subject	29,13				
ANOVA table	SS	DF	MS	F (DFn, DFd)	P value
network	0,2755	3	0,09184	F (1,018, 9,158) = 3,595	P=0,0895
shuffling	0,7416	1	0,7416	F (1,000, 9,000) = 189,2	P<0,0001
network x shuffling	0,1755	3	0,05851	F (1,885, 16,96) = 88,19	P<0,0001
Subject x network	0,6899	27	0,02555		
Subject x shuffling	0,03527	9	0,003919		
Subject	0,7958	9	0,08842		
Residual	0,01791	27	0,0006635		
.					

**Bonferroni's multiple comparisons test**	Mean Diff,	95,00% CI	Sign.?	Summary	Adj. P Value	
granule full:non-shuffled vs. granule full:shuffled	0,2713	0,1763 to	Yes	****	<0,0001	
granule full:non-shuffled vs. granule no-feedforward:non-shuffled	0,06366	0,02850 to	Yes	***	0,0007	
granule full:non-shuffled vs. granule no-feedforward:shuffled	0,3636	0,2581 to	Yes	****	<0,0001	
granule full:non-shuffled vs. granule no-feedback:non-shuffled	0,2491	0,04597 to	Yes	*	0,0127	
granule full:non-shuffled vs. granule no-feedback:shuffled	0,3539	0,1594 to	Yes	***	0,0006	
granule full:non-shuffled vs. granule disinhibited:non-shuffled	0,1723	-0,1735 to	No	ns	>0,9999	
granule full:non-shuffled vs. granule disinhibited:shuffled	0,2665	-0,08724 to	No	ns	0,2603	
granule full:shuffled vs. granule no-feedforward:non-shuffled	-0,2076	-0,2964 to -	Yes	****	<0,0001	
granule full:shuffled vs. granule no-feedforward:shuffled	0,09238	0,05371 to	Yes	****	<0,0001	
granule full:shuffled vs. granule no-feedback:non-shuffled	-0,02216	-0,2055 to	No	ns	>0,9999	
granule full:shuffled vs. granule no-feedback:shuffled	0,08264	-0,07613 to	No	ns	>0,9999	
granule full:shuffled vs. granule disinhibited:non-shuffled	-0,09894	-0,4250 to	No	ns	>0,9999	
granule full:shuffled vs. granule disinhibited:shuffled	-0,004749	-0,3317 to	No	ns	>0,9999	
granule no-feedforward:non-shuffled vs. granule no-	0,3000	0,2010 to	Yes	****	<0,0001	
granule no-feedforward:non-shuffled vs. granule no-	0,1854	-0,008490 to	No	ns	0,0663	
granule no-feedforward:non-shuffled vs. granule no-	0,2902	0,1033 to	Yes	**	0,0022	
granule no-feedforward:non-shuffled vs. granule disinhibited:non-	0,1086	-0,2289 to	No	ns	>0,9999	
granule no-feedforward:non-shuffled vs. granule	0,2028	-0,1427 to	No	ns	0,8485	
granule no-feedforward:shuffled vs. granule no-feedback:non-	-0,1145	-0,2672 to	No	ns	0,2663	
granule no-feedforward:shuffled vs. granule no-feedback:shuffled	-0,009742	-0,1356 to	No	ns	>0,9999	
granule no-feedforward:shuffled vs. granule disinhibited:non-	-0,1913	-0,4840 to	No	ns	0,5263	
granule no-feedforward:shuffled vs. granule disinhibited:shuffled	-0,09713	-0,3911 to	No	ns	>0,9999	
granule no-feedback:non-shuffled vs. granule no-	0,1048	0,06043 to	Yes	****	<0,0001	
granule no-feedback:non-shuffled vs. granule disinhibited:non-	-0,07678	-0,2250 to	No	ns	>0,9999	
granule no-feedback:non-shuffled vs. granule disinhibited:shuffled	0,01741	-0,1398 to	No	ns	>0,9999	
granule no-feedback:shuffled vs. granule disinhibited:non-shuffled	-0,1816	-0,3527 to -	Yes	*	0,0341	
granule no-feedback:shuffled vs. granule disinhibited:shuffled	-0,08739	-0,2618 to	No	ns	>0,9999	
granule disinhibited:non-shuffled vs. granule disinhibited:shuffled	0,09419	0,05128 to	Yes	***	0,0001	

Supplementary Table 23. Statistics for Supplementary Fig. 8c – Positional rate information analysis (smoothing window = 12cm)

**Suppl. Fig. 8b phase**	Positional phase information analysis, 12cm smoothing				
**Two-way RM ANOVA**	Matching: Both factors				
Assume sphericity?	No				
Alpha	0,05				
Source of Variation	% of total variation	P value	P value summary	Significant?	Geisser-
network	85,42	<0,0001	****	Yes	0,4308
shuffling	8,544	<0,0001	****	Yes	1,000
network x shuffling	3,441	<0,0001	****	Yes	0,5129
Subject x network	0,6776				
Subject x shuffling	0,7267				
Subject	0,7732				
ANOVA table	SS	DF	MS	F (DFn, DFd)	P value
network	1,595	3	0,5317	F (1,292, 11,63) = 1135	P<0,0001
shuffling	0,1595	1	0,1595	F (1,000, 9,000) = 105,8	P<0,0001
network x shuffling	0,06425	3	0,02142	F (1,539, 13,85) = 74,69	P<0,0001
Subject x network	0,01265	27	0,0004686		
Subject x shuffling	0,01357	9	0,001508		
Subject	0,01444	9	0,001604		
Residual	0,007742	27	0,0002867		
.					

**Bonferroni's multiple comparisons test**	Mean Diff,	95,00% CI	Sign.?	Summary	Adj. P Value	
granule full:non-shuffled vs. granule full:shuffled	0,04547	0,003494 to	Yes	*	0,0297	
granule full:non-shuffled vs. granule no-feedforward:non-shuffled	0,04269	0,02122 to	Yes	***	0,0003	
granule full:non-shuffled vs. granule no-feedforward:shuffled	0,07215	0,02795 to	Yes	**	0,0015	
granule full:non-shuffled vs. granule no-feedback:non-shuffled	-0,2697	-0,3078 to -	Yes	****	<0,0001	
granule full:non-shuffled vs. granule no-feedback:shuffled	-0,1599	-0,1973 to -	Yes	****	<0,0001	
granule full:non-shuffled vs. granule disinhibited:non-shuffled	-0,3498	-0,4006 to -	Yes	****	<0,0001	
granule full:non-shuffled vs. granule disinhibited:shuffled	-0,1772	-0,2443 to -	Yes	****	<0,0001	
granule full:shuffled vs. granule no-feedforward:non-shuffled	-0,002781	-0,02690 to	No	ns	>0,9999	
granule full:shuffled vs. granule no-feedforward:shuffled	0,02668	0,01465 to	Yes	***	0,0001	
granule full:shuffled vs. granule no-feedback:non-shuffled	-0,3152	-0,3729 to -	Yes	****	<0,0001	
granule full:shuffled vs. granule no-feedback:shuffled	-0,2054	-0,2204 to -	Yes	****	<0,0001	
granule full:shuffled vs. granule disinhibited:non-shuffled	-0,3952	-0,4316 to -	Yes	****	<0,0001	
granule full:shuffled vs. granule disinhibited:shuffled	-0,2227	-0,2616 to -	Yes	****	<0,0001	
granule no-feedforward:non-shuffled vs. granule no-	0,02946	0,002560 to	Yes	*	0,0276	
granule no-feedforward:non-shuffled vs. granule no-	-0,3124	-0,3595 to -	Yes	****	<0,0001	
granule no-feedforward:non-shuffled vs. granule no-	-0,2026	-0,2229 to -	Yes	****	<0,0001	
granule no-feedforward:non-shuffled vs. granule disinhibited:non-	-0,3924	-0,4343 to -	Yes	****	<0,0001	
granule no-feedforward:non-shuffled vs. granule	-0,2199	-0,2689 to -	Yes	****	<0,0001	
granule no-feedforward:shuffled vs. granule no-feedback:non-	-0,3419	-0,3988 to -	Yes	****	<0,0001	
granule no-feedforward:shuffled vs. granule no-feedback:shuffled	-0,2321	-0,2469 to -	Yes	****	<0,0001	
granule no-feedforward:shuffled vs. granule disinhibited:non-	-0,4219	-0,4632 to -	Yes	****	<0,0001	
granule no-feedforward:shuffled vs. granule disinhibited:shuffled	-0,2494	-0,2913 to -	Yes	****	<0,0001	
granule no-feedback:non-shuffled vs. granule no-	0,1098	0,05360 to	Yes	***	0,0004	
granule no-feedback:non-shuffled vs. granule disinhibited:non-	-0,08004	-0,1383 to -	Yes	**	0,0056	
granule no-feedback:non-shuffled vs. granule disinhibited:shuffled	0,09249	0,0001131 to	Yes	*	0,0496	
granule no-feedback:shuffled vs. granule disinhibited:non-shuffled	-0,1898	-0,2347 to -	Yes	****	<0,0001	
granule no-feedback:shuffled vs. granule disinhibited:shuffled	-0,01732	-0,05758 to	No	ns	>0,9999	
granule disinhibited:non-shuffled vs. granule disinhibited:shuffled	0,1725	0,1141 to	Yes	****	<0,0001	

Supplementary Table 24. Statistics for Supplementary Fig. 8c – Positional rate information analysis (smoothing window = 12cm)

**Suppl. Fig. 8c rate**	Positional rate information analysis, 6cm smoothing				
**Two-way RM ANOVA**	Matching: Both factors				
Assume sphericity?	No				
Alpha	0,05				
Source of Variation	% of total variation	P value	P value summary	Significant?	Geisser-
network	49,45	<0,0001	****	Yes	0,3479
shuffling	27,06	<0,0001	****	Yes	1,000
network x shuffling	9,485	<0,0001	****	Yes	0,7323
Subject x network	3,747				
Subject x shuffling	0,4350				
Subject	9,583				
ANOVA table	SS	DF	MS	F (DFn, DFd)	P value
network	4,415	3	1,472	F (1,044, 9,392) = 118,8	P<0,0001
shuffling	2,415	1	2,415	F (1,000, 9,000) = 559,7	P<0,0001
network x shuffling	0,8468	3	0,2823	F (2,197, 19,77) = 354,3	P<0,0001
Subject x network	0,3345	27	0,01239		
Subject x shuffling	0,03884	9	0,004315		
Subject	0,8556	9	0,09507		
Residual	0,02151	27	0,0007967		
.					

**Bonferroni's multiple comparisons test**	Mean Diff,	95,00% CI	Sign.?	Summary	Adj. P Value	
granule full:non-shuffled vs. granule full:shuffled	0,5254	0,4163 to	Yes	****	<0,0001	
granule full:non-shuffled vs. granule no-feedforward:non-shuffled	0,1565	0,1043 to	Yes	****	<0,0001	
granule full:non-shuffled vs. granule no-feedforward:shuffled	0,7358	0,6447 to	Yes	****	<0,0001	
granule full:non-shuffled vs. granule no-feedback:non-shuffled	0,6856	0,5606 to	Yes	****	<0,0001	
granule full:non-shuffled vs. granule no-feedback:shuffled	0,8333	0,7055 to	Yes	****	<0,0001	
granule full:non-shuffled vs. granule disinhibited:non-shuffled	0,7757	0,5636 to	Yes	****	<0,0001	
granule full:non-shuffled vs. granule disinhibited:shuffled	0,9133	0,6936 to 1,133	Yes	****	<0,0001	
granule full:shuffled vs. granule no-feedforward:non-shuffled	-0,3690	-0,4750 to -	Yes	****	<0,0001	
granule full:shuffled vs. granule no-feedforward:shuffled	0,2104	0,1591 to	Yes	****	<0,0001	
granule full:shuffled vs. granule no-feedback:non-shuffled	0,1602	0,01085 to	Yes	*	0,0317	
granule full:shuffled vs. granule no-feedback:shuffled	0,3079	0,1773 to	Yes	****	<0,0001	
granule full:shuffled vs. granule disinhibited:non-shuffled	0,2503	0,001741 to	Yes	*	0,0478	
granule full:shuffled vs. granule disinhibited:shuffled	0,3879	0,1431 to	Yes	**	0,0019	
granule no-feedforward:non-shuffled vs. granule no-	0,5793	0,4767 to	Yes	****	<0,0001	
granule no-feedforward:non-shuffled vs. granule no-	0,5291	0,3873 to	Yes	****	<0,0001	
granule no-feedforward:non-shuffled vs. granule no-	0,6769	0,5288 to	Yes	****	<0,0001	
granule no-feedforward:non-shuffled vs. granule disinhibited:non-	0,6193	0,3874 to	Yes	****	<0,0001	
granule no-feedforward:non-shuffled vs. granule	0,7569	0,5144 to	Yes	****	<0,0001	
granule no-feedforward:shuffled vs. granule no-feedback:non-	-0,05019	-0,1789 to	No	ns	>0,9999	
granule no-feedforward:shuffled vs. granule no-feedback:shuffled	0,09752	-0,01421 to	No	ns	0,1148	
granule no-feedforward:shuffled vs. granule disinhibited:non-	0,03992	-0,1869 to	No	ns	>0,9999	
granule no-feedforward:shuffled vs. granule disinhibited:shuffled	0,1776	-0,04384 to	No	ns	0,1858	
granule no-feedback:non-shuffled vs. granule no-	0,1477	0,1094 to	Yes	****	<0,0001	
granule no-feedback:non-shuffled vs. granule disinhibited:non-	0,09011	-0,01448 to	No	ns	0,1239	
granule no-feedback:non-shuffled vs. granule disinhibited:shuffled	0,2278	0,1218 to	Yes	***	0,0002	
granule no-feedback:shuffled vs. granule disinhibited:non-shuffled	-0,05760	-0,1802 to	No	ns	>0,9999	
granule no-feedback:shuffled vs. granule disinhibited:shuffled	0,08004	-0,03649 to	No	ns	0,4157	
granule disinhibited:non-shuffled vs. granule disinhibited:shuffled	0,1376	0,09564 to	Yes	****	<0,0001	

Supplementary Table 25. Statistics for Supplementary Fig. 8b – Positional rate information analysis (smoothing window = 6 cm)

Suppl. Fig. 8c phase	Positional phase information analysis, 6cm smoothing				
**Two-way RM ANOVA**	Matching: Both factors				
Assume sphericity?	No				
Alpha	0,05				
Source of Variation	% of total variation	P value	P value summary	Significant?	Geisser-
network	85,42	<0,0001	****	Yes	0,4308
shuffling	8,544	<0,0001	****	Yes	1,000
network x shuffling	3,441	<0,0001	****	Yes	0,5129
Subject x network	0,6776				
Subject x shuffling	0,7267				
Subject	0,7732				
ANOVA table	SS	DF	MS	F (DFn, DFd)	P value
network	1,595	3	0,5317	F (1,292, 11,63) = 1135	P<0,0001
shuffling	0,1595	1	0,1595	F (1,000, 9,000) = 105,8	P<0,0001
network x shuffling	0,06425	3	0,02142	F (1,539, 13,85) = 74,69	P<0,0001
Subject x network	0,01265	27	0,0004686		
Subject x shuffling	0,01357	9	0,001508		
Subject	0,01444	9	0,001604		
Residual	0,007742	27	0,0002867		
.					

**Bonferroni's multiple comparisons test**	Mean Diff,	95,00% CI	Sign.?	Summary	Adj. P Value	
granule full:non-shuffled vs. granule full:shuffled	0,04547	0,003494 to	Yes	*	0,0297	
granule full:non-shuffled vs. granule no-feedforward:non-shuffled	0,04269	0,02122 to	Yes	***	0,0003	
granule full:non-shuffled vs. granule no-feedforward:shuffled	0,07215	0,02795 to	Yes	**	0,0015	
granule full:non-shuffled vs. granule no-feedback:non-shuffled	-0,2697	-0,3078 to -	Yes	****	<0,0001	
granule full:non-shuffled vs. granule no-feedback:shuffled	-0,1599	-0,1973 to -	Yes	****	<0,0001	
granule full:non-shuffled vs. granule disinhibited:non-shuffled	-0,3498	-0,4006 to -	Yes	****	<0,0001	
granule full:non-shuffled vs. granule disinhibited:shuffled	-0,1772	-0,2443 to -	Yes	****	<0,0001	
granule full:shuffled vs. granule no-feedforward:non-shuffled	-0,002781	-0,02690 to	No	ns	>0,9999	
granule full:shuffled vs. granule no-feedforward:shuffled	0,02668	0,01465 to	Yes	***	0,0001	
granule full:shuffled vs. granule no-feedback:non-shuffled	-0,3152	-0,3729 to -	Yes	****	<0,0001	
granule full:shuffled vs. granule no-feedback:shuffled	-0,2054	-0,2204 to -	Yes	****	<0,0001	
granule full:shuffled vs. granule disinhibited:non-shuffled	-0,3952	-0,4316 to -	Yes	****	<0,0001	
granule full:shuffled vs. granule disinhibited:shuffled	-0,2227	-0,2616 to -	Yes	****	<0,0001	
granule no-feedforward:non-shuffled vs. granule no-	0,02946	0,002560 to	Yes	*	0,0276	
granule no-feedforward:non-shuffled vs. granule no-	-0,3124	-0,3595 to -	Yes	****	<0,0001	
granule no-feedforward:non-shuffled vs. granule no-	-0,2026	-0,2229 to -	Yes	****	<0,0001	
granule no-feedforward:non-shuffled vs. granule disinhibited:non-	-0,3924	-0,4343 to -	Yes	****	<0,0001	
granule no-feedforward:non-shuffled vs. granule	-0,2199	-0,2689 to -	Yes	****	<0,0001	
granule no-feedforward:shuffled vs. granule no-feedback:non-	-0,3419	-0,3988 to -	Yes	****	<0,0001	
granule no-feedforward:shuffled vs. granule no-feedback:shuffled	-0,2321	-0,2469 to -	Yes	****	<0,0001	
granule no-feedforward:shuffled vs. granule disinhibited:non-	-0,4219	-0,4632 to -	Yes	****	<0,0001	
granule no-feedforward:shuffled vs. granule disinhibited:shuffled	-0,2494	-0,2913 to -	Yes	****	<0,0001	
granule no-feedback:non-shuffled vs. granule no-	0,1098	0,05360 to	Yes	***	0,0004	
granule no-feedback:non-shuffled vs. granule disinhibited:non-	-0,08004	-0,1383 to -	Yes	**	0,0056	
granule no-feedback:non-shuffled vs. granule disinhibited:shuffled	0,09249	0,0001131 to	Yes	*	0,0496	
granule no-feedback:shuffled vs. granule disinhibited:non-shuffled	-0,1898	-0,2347 to -	Yes	****	<0,0001	
granule no-feedback:shuffled vs. granule disinhibited:shuffled	-0,01732	-0,05758 to	No	ns	>0,9999	
granule disinhibited:non-shuffled vs. granule disinhibited:shuffled	0,1725	0,1141 to	Yes	****	<0,0001	

Supplementary Table 26. Statistics for Supplementary Fig. 8b – Positional phase information analysis (smoothing window 6 cm)

Suppl. Fig. 8b	Grid rate code (12cm smooting)
Column B	S
vs.	vs,
Column A	ns
**Paired t test**	
Test not possible because all values are identical	

Suppl. Fig. 8b	Grid phase code (12cm smoothing)
Column B	sm12_s
vs.	vs,
Column A	sm12_ns
**Paired t test**	
P value	<0,0001
Significantly different (P <	Yes
One- or two-tailed P value?	Two-tailed
t, df	t=14,93, df=9
Number of pairs	10
How big is the difference?	
Mean of differences (B - A)	-0,07938
SD of differences	0,01682
SEM of differences	0,005318
95% confidence interval	-0,09140 to -0,06735
R squared (partial eta	0,9612
How effective was the	
Correlation coefficient (r)	0,6467
P value (one tailed)	0,0217
P value summary	*

Suppl. Fig. 8c	Grid rate code (6cm smoothing)
Column B	s
vs.	vs,
Column A	ns
**Paired t test**	
Test not possible because all values are identical	

Suppl. Fig. 8c	Grid phase code (6cm smoothing)
Column B	sm6_s
vs.	vs,
Column A	sm6_ns
**Paired t test**	
<0,0001	<0,0001
P value summary	****
Significantly different (P <	Yes
One- or two-tailed P value?	Two-tailed
t, df	t=20,63, df=9
Number of pairs	10
How big is the difference?	
Mean of differences (B - A)	-0,08023
SD of differences	0,01230
SEM of differences	0,003888
95% confidence interval	-0,08903 to -0,07144
R squared (partial eta	0,9793
How effective was the	
Correlation coefficient (r)	0,7986
P value (one tailed)	0,0028
P value summary	**

Supplementary Table 27. Statistics for Fig. 8b,c grid cell data – mean rate or phase info in grid population for non-shuffeld vs. shuffeld networks

Suppl. Fig. 11a	Positional rate info analysis for only the first half of theta				
Two-way RM ANOVA	Matching: Both factors				
Assume sphericity?	No				
Alpha	0,05				
Source of Variation	% of total variation	P value	P value summary	Significant?	Geisser-
shuffling	48,92	<0,0001	****	Yes	1,000
network	36,23	<0,0001	****	Yes	1,000
shuffling x network	3,400	<0,0001	****	Yes	1,000
grid_seed x shuffling	0,9145				
grid_seed x network	1,329				
grid_seed	8,998				
ANOVA table	SS	DF	MS	F (DFn, DFd)	P value
shuffling	2,110	1	2,110	F (1,000, 9,000) = 481,4	P<0,0001
network	1,563	1	1,563	F (1,000, 9,000) = 245,4	P<0,0001
shuffling x network	0,1466	1	0,1466	F (1,000, 9,000) = 143,8	P<0,0001
grid_seed x shuffling	0,03945	9	0,004383		
grid_seed x network	0,05732	9	0,006369		
grid_seed	0,3881	9	0,04312		
Residual	0,009179	9	0,001020		
.					

**Post-hoc test**								
Number of families	1							
Number of comparisons per	3							
Alpha	0,05							
Dunnett's multiple comparisons	Mean Diff,	95,00% CI of diff,	Below	Summary	Adjusted P			
ns:full vs. ns:no-fb	0,5164	0,4541 to 0,5787	Yes	****	<0,0001			
ns:full vs. s:full	0,5804	0,5043 to 0,6565	Yes	****	<0,0001			
ns:full vs. s:no-fb	0,8547	0,7540 to 0,9553	Yes	****	<0,0001			
Test details	Mean 1	Mean 2	Mean Diff,	SE of diff,	N1	N2	q	DF
ns:full vs. ns:no-fb	2,606	2,090	0,5164	0,02215	10	10	23,31	9,000
ns:full vs. s:full	2,606	2,026	0,5804	0,02706	10	10	21,45	9,000
ns:full vs. s:no-fb	2,606	1,752	0,8547	0,03578	10	10	23,88	9,000

Supplementary Table 28. Statistics for Supplementary Fig. 11a – positional rate information analysis for only early theta (0-pi)

**Suppl. Fig. 11b**	Positional phase info analysis for only the first half of theta				
Two-way RM ANOVA	Matching: Both factors				
Assume sphericity?	No				
Alpha	0,05				
Source of Variation	% of total variation	P value	P value summary	Significant?	Geisser-
shuffling	30,86	<0,0001	****	Yes	1,000
network	46,71	<0,0001	****	Yes	1,000
shuffling x network	2,233	0,0007	***	Yes	1,000
grid_seed x shuffling	2,331				
grid_seed x network	7,992				
grid_seed	9,086				
ANOVA table	SS	DF	MS	F (DFn, DFd)	P value
shuffling	0,001791	1	0,001791	F (1,000, 9,000) = 119,1	P<0,0001
network	0,002711	1	0,002711	F (1,000, 9,000) = 52,60	P<0,0001
shuffling x network	0,0001296	1	0,0001296	F (1,000, 9,000) = 25,54	P=0,0007
grid_seed x shuffling	0,0001353	9	1,503e-005		
grid_seed x network	0,0004638	9	5,153e-005		
grid_seed	0,0005273	9	5,859e-005		
Residual	4,567e-005	9	5,075e-006		
.					

**Post-hoc test**								
Number of families	1							
Number of comparisons per	3							
Alpha	0,05							
Dunnett's multiple comparisons	Mean Diff,	95,00% CI of diff,	Below	Summary	Adjusted P			
ns:full vs. ns:no-fb	-0,02007	-0,02795 to -0,01218	Yes	***	0,0002			
ns:full vs. s:full	0,009782	0,006549 to 0,01301	Yes	****	<0,0001			
ns:full vs. s:no-fb	-0,003083	-0,009498 to	No	ns	0,4321			
Test details	Mean 1	Mean 2	Mean Diff,	SE of diff,	N1	N2	q	DF
ns:full vs. ns:no-fb	0,01152	0,03159	-0,02007	0,002803	10	10	7,159	9,000
ns:full vs. s:full	0,01152	0,001741	0,009782	0,001150	10	10	8,509	9,000
ns:full vs. s:no-fb	0,01152	0,01461	-0,003083	0,002281	10	10	1,352	9,000

Supplementary Table 29. Statistics for Supplementary Fig. 11b – positional phase information analysis for only early theta (0-pi)

**Suppl. Fig. 13d**	GC rates	**Suppl. Fig. 13d**	CA3 Pyr. rates
Column B	noFB	Column B	noFB
vs.	vs,	vs.	vs,
Column A	full	Column A	full
Paired t test		Paired t test	
P value	0,0230	P value	0,0006
Mult. Comp. corr. P value	0.0690	Mult. Comp. corr. P value	0.0018
P value summary	ns.	P value summary	**
Significant (P < 0.05)?	No	Significantly different (P <	Yes
One- or two-tailed P value?	Two-tailed	One- or two-tailed P value?	Two-tailed
t, df	t=2,531127, df=15	t, df	t=4,322766, df=15
Number of pairs	16	Number of pairs	16
How big is the difference?		How big is the difference?	
Mean of differences (B - A)	-0,007707813	Mean of differences (B - A)	-0,2816563
SD of differences	0,01218084	SD of differences	0,2606260
SEM of differences	0,003045209	SEM of differences	0,06515650
95% confidence interval	-0,01419852 to -	95% confidence interval	-0,4205341 to -0,1427785
R squared (partial eta	0,2992817	R squared (partial eta	0,5547152
How effective was pairing?		How effective was pairing?	
Correlation coefficient (r)	0,8942565	Correlation coefficient (r)	0,9635988
P value (one tailed)	<0,0001	P value (one tailed)	<0,0001
P value summary	****	P value summary	****

**Suppl. Fig. 13d**	CA3 Int. rates		
Column B	noFB		
vs.	vs,		
Column A	full		
Paired t test			
Mult. Comp. corr. P value	>0,99		
P value summary	ns		
Significantly different (P <	No		
One- or two-tailed P value?	Two-tailed		
t, df	t=0,8695451, df=15		
Number of pairs	16		
How big is the difference?			
Mean of differences (B - A)	0,8368225		
SD of differences	3,849473		
SEM of differences	0,9623682		
95% confidence interval	-1,214417 to 2,888062		
R squared (partial eta	0,04798829		
How effective was pairing?			
Correlation coefficient (r)	0,8649341		
P value (one tailed)	<0,0001		
P value summary	****		

Supplementary Table 30. Statistics for Supplementary Fig. 13 – Mean rates for extended CA3 model

Suppl. Fig. 13f	Mean weight increase		Suppl. Fig. 13h	Mean weight/rate
Column B	noFB		Column B	noFB
vs.	vs,		vs.	vs,
Column A	full		Column A	full
Paired t test			Paired t test	
P value	<0,0001		P value	<0,0001
P value summary	****		P value summary	****
Significantly different (P <	Yes		Significantly different (P <	Yes
One- or two-tailed P value?	Two-tailed		One- or two-tailed P value?	Two-tailed
t, df	t=10,09797, df=15		t, df	t=19,60410, df=15
Number of pairs	16		Number of pairs	16
How big is the difference?			How big is the difference?	
Mean of differences (B - A)	-0,01630838		Mean of differences (B - A)	-0,004324750
SD of differences	0,006460059		SD of differences	0,0008824175
SEM of differences	0,001615015		SEM of differences	0,0002206044
95% confidence interval	-0,01975070 to -0,01286605		95% confidence interval	-0,004794957 to -
R squared (partial eta	0,8717610		R squared (partial eta	0,9624362
How effective was the			How effective was the	
Correlation coefficient (r)	0,9623381		Correlation coefficient (r)	0,9598502
P value (one tailed)	<0,0001		P value (one tailed)	<0,0001
P value summary	****		P value summary	****
Was the pairing significantly	Yes		Was the pairing	Yes

Supplementary Table 31. Statistics for Supplementary Fig. 13 – Mean weights & weights/rates for extended CA3 model

Supplementary References

1. Solstad, T., Moser, E. I. & Einevoll, G. T. From grid cells to place cells: A mathematical model. *Hippocampus* **16**, 1026–1031 (2006).
2. Bush, D. & Burgess, N. Advantages and detection of phase coding in the absence of rhythmicity. *Hippocampus* **30**, 745–762 (2020).
3. Stensola, H. *et al.* The entorhinal grid map is discretized. *Nature* **492**, 72–78 (2012).
4. Ebbesen, C. L. *et al.* Cell Type-Specific Differences in Spike Timing and Spike Shape in the Rat Parasubiculum and Superficial Medial Entorhinal Cortex. *Cell Rep.* **16**, 1005–1015 (2016).
5. Reifenstein, E. T. *et al.* Cell-Type Specific Phase Precession in Layer II of the Medial Entorhinal Cortex. *J. Neurosci.* **36**, 2283–2288 (2016).
6. Mizuseki, K., Sirota, A., Pastalkova, E. & Buzs?ki, G. Theta Oscillations Provide Temporal Windows for Local Circuit Computation in the Entorhinal-Hippocampal Loop. *Neuron* **64**, 267–280 (2009).
7. Rowland, D. C. *et al.* Functional properties of stellate cells in medial entorhinal cortex layer II. *eLife* **7**, e36664 (2018).
8. Ray, S. *et al.* Grid-Layout and Theta-Modulation of Layer 2 Pyramidal Neurons in Medial Entorhinal Cortex. *Science* **343**, 891–896 (2014).
9. Pernía-Andrade, A. J. & Jonas, P. Theta-gamma-modulated synaptic currents in hippocampal granule cells in vivo define a mechanism for network oscillations. *Neuron* **81**, 140–52 (2014).
10. Olypher, A. V., Lánský, P., Muller, R. U. & Fenton, A. A. Quantifying location-specific information in the discharge of rat hippocampal place cells. *J. Neurosci. Methods* **127**, 123–135 (2003).
11. Tingley, D. & Buzsáki, G. Transformation of a Spatial Map across the Hippocampal-Lateral Septal Circuit. *Neuron* **98**, 1229-1242.e5 (2018).
12. Toth, K., Suares, G., Lawrence, J. J., Philips-Tansey, E. & McBain, C. J. Differential mechanisms of transmission at three types of mossy fiber synapse. *J. Neurosci. Off. J. Soc. Neurosci.* **20**, 8279–89 (2000).
13. Henze, D. A., Wittner, L. & Buzsáki, G. Single granule cells reliably discharge targets in the hippocampal CA3 network in vivo. *Nat. Neurosci.* **5**, 790–5 (2002).

14. Schoenfeld, G., Carta, S., Rupprecht, P., Ayaz, A. & Helmchen, F. In Vivo Calcium Imaging of CA3 Pyramidal Neuron Populations in Adult Mouse Hippocampus. *eNeuro* **8**, (2021).
