# Supplementary material for: Phase information is conserved in sparse, synchronous population-rate-codes via phase-to-rate recoding
Source: Nat Commun. 2023 Sep 30;14:6106. doi: 10.1038/s41467-023-41803-8 (PMC10543394; doi:10.1038/s41467-023-41803-8)
Supplement: Supplementary file 3 — Reporting Summary [file 41467_2023_41803_MOESM3_ESM.pdf]

nature portfolio

Corresponding author(s): Daniel Müller-Komorowska, Oliver Braganza

Last updated by author(s): 25.08.2023

Reporting Summary

Nature Portfolio wishes to improve the reproducibility of the work that we publish. This form provides structure for consistency and transparency in reporting. For further information on Nature Portfolio policies, see our Editorial Policies and the Editorial Policy Checklist.

Statistics

For all statistical analyses, confirm that the following items are present in the figure legend, table legend, main text, or Methods section.

n/a	Confirmed	
☐	☒	The exact sample size (n) for each experimental group/condition, given as a discrete number and unit of measurement
☐	☒	A statement on whether measurements were taken from distinct samples or whether the same sample was measured repeatedly
☐	☒	The statistical test(s) used AND whether they are one- or two-sided<br>*Only common tests should be described solely by name; describe more complex techniques in the Methods section.*
☐	☒	A description of all covariates tested
☐	☒	A description of any assumptions or corrections, such as tests of normality and adjustment for multiple comparisons
☐	☒	A full description of the statistical parameters including central tendency (e.g. means) or other basic estimates (e.g. regression coefficient) AND variation (e.g. standard deviation) or associated estimates of uncertainty (e.g. confidence intervals)
☐	☒	For null hypothesis testing, the test statistic (e.g. F, t, r) with confidence intervals, effect sizes, degrees of freedom and P value noted<br>*Give P values as exact values whenever suitable.*
☒	☐	For Bayesian analysis, information on the choice of priors and Markov chain Monte Carlo settings
☐	☒	For hierarchical and complex designs, identification of the appropriate level for tests and full reporting of outcomes
☐	☒	Estimates of effect sizes (e.g. Cohen's d, Pearson's r), indicating how they were calculated

Our web collection on statistics for biologists contains articles on many of the points above.

Software and code

Policy information about availability of computer code

Data collection	No data collection software was used. All data were simulated.
Data analysis	All code required to reproduce the present data and figures is available at https://doi.org/10.5281/zenodo.8278084 or https://github.com/barisckuru/phase-to-rate. Our models relied on Python 3.9.12 and standard software packages, namely NumPy 1.22.4 82, elephant 0.11.1 71, neo 0.10.2 83, seaborn 0.11.2 84, PyTorch 1.12.1 74, as well as the NEURON 8.0.0-2-g0e9a0517+ 85 and Brian2 2.575 modeling environments. Statistical analysis was performed using GraphPad Prism 4 or 7.

For manuscripts utilizing custom algorithms or software that are central to the research but not yet described in published literature, software must be made available to editors and reviewers. We strongly encourage code deposition in a community repository (e.g. GitHub). See the Nature Portfolio guidelines for submitting code & software for further information.

Data

Policy information about availability of data

All manuscripts must include a data availability statement. This statement should provide the following information, where applicable:

- Accession codes, unique identifiers, or web links for publicly available datasets
- A description of any restrictions on data availability
- For clinical datasets or third party data, please ensure that the statement adheres to our policy

The pre-simulated spike data generated in this study have been deposited in zenodo under accession code 10.5281/zenodo.8280121 (https://doi.org/10.5281/zenodo.8280121). The processed data underlying the figures are provided with this paper as Source Data file.

Research involving human participants, their data, or biological material

Policy information about studies with human participants or human data. See also policy information about sex, gender (identity/presentation), and sexual orientation and race, ethnicity and racism.

Reporting on sex and gender	n/a
Reporting on race, ethnicity, or other socially relevant groupings	n/a
Population characteristics	n/a
Recruitment	n/a
Ethics oversight	n/a

Note that full information on the approval of the study protocol must also be provided in the manuscript.

Field-specific reporting

Please select the one below that is the best fit for your research. If you are not sure, read the appropriate sections before making your selection.

☒ Life sciences ☐ Behavioural & social sciences ☐ Ecological, evolutionary & environmental sciences

For a reference copy of the document with all sections, see nature.com/documents/nr-reporting-summary-flat.pdf

Life sciences study design

All studies must disclose on these points even when the disclosure is negative.

Sample size	As no prior estimates of effect size were available, no predetermination of sample size was made. We chose a sample sizes of 10 (Fig.1-3) or 30 (Fig.4) since we were interested in large effects. The sample sizes are unambiguously sufficient to detect the described effects, because they consistently detected the effect (at least 7 times) across multiple readout measures (information & decoding analyses) and robustness analyses (insufficient sample sizes would have yielded numerous false negative results). Sample sizes for Supplementary analyses varied between 10 and 20 due to time constraints, but are also unambiguously sufficient, because they consistently reported the effect.
Data exclusions	For analyses with 'controlled GC rates', we excluded all data for which mean GC rates were not in the range of 0.2 to 0.3Hz. Otherwise no data was excluded.
Replication	The central finding (phase-to-rate recoding) was replicated at least 4 times during robustness analyses (Fig.2, supplementary Figs. 5-7). Additionally, Figs. 3,4 and Supplementary Figs. 8,11 represent conceptual replications of the central finding. All replications confirmed the original finding.
Randomization	Reproducible randomization was achieved by setting the seed of a random python process (numpy.random).
Blinding	Not applicable (i.e. inherently given), since all data, independent of condition, was automatically processed by the same code without supervision or interference.

Reporting for specific materials, systems and methods

We require information from authors about some types of materials, experimental systems and methods used in many studies. Here, indicate whether each material, system or method listed is relevant to your study. If you are not sure if a list item applies to your research, read the appropriate section before selecting a response.

Materials & experimental systems

n/a	Involved in the study
☒	☐ Antibodies
☒	☐ Eukaryotic cell lines
☒	☐ Palaeontology and archaeology
☒	☐ Animals and other organisms
☒	☐ Clinical data
☒	☐ Dual use research of concern
☒	☐ Plants

Methods

n/a	Involved in the study
☒	☐ ChIP-seq
☒	☐ Flow cytometry
☒	☐ MRI-based neuroimaging
